# Accurate long-read de novo assembly evaluation with Inspector

**DOI:** 10.1186/s13059-021-02527-4

**Published:** 2021-11-14

**Authors:** Yu Chen, Yixin Zhang, Amy Y. Wang, Min Gao, Zechen Chong

**Affiliations:** 1 Department of Genetics, Heersink School of Medicine, University of Alabama at Birmingham, Birmingham, AL 35294 USA; 2 Informatics Institute, Heersink School of Medicine, University of Alabama at Birmingham, Birmingham, AL 35294 USA; 3 Department of Computer Science, College of Arts and Sciences, University of Alabama at Birmingham, Birmingham, AL 35294 USA; 4 Department of Medicine, Division of General Internal Medicine, Heersink School of Medicine, University of Alabama at Birmingham, Birmingham, AL 35294 USA; 5 Department of Medicine, Division of Cardiovascular Disease, Heersink School of Medicine, University of Alabama at Birmingham, AL 35233 Birmingham, USA

**Keywords:** De novo assembly, Long reads, Assembly evaluation, Assembly error, Genome assembly

## Abstract

Long-read de novo genome assembly continues to advance rapidly. However, there is a lack of effective tools to accurately evaluate the assembly results, especially for structural errors. We present Inspector, a reference-free long-read de novo assembly evaluator which faithfully reports types of errors and their precise locations. Notably, Inspector can correct the assembly errors based on consensus sequences derived from raw reads covering erroneous regions. Based on in silico and long-read assembly results from multiple long-read data and assemblers, we demonstrate that in addition to providing generic metrics, Inspector can accurately identify both large-scale and small-scale assembly errors.

**Supplementary Information:**

The online version contains supplementary material available at 10.1186/s13059-021-02527-4.

## Background

Whole-genome de novo assembly is essential for investigating species without reference genomes and is critical for characterizing the full spectrum of genetic variants for species with a reference genome [1–8]. With the advancement of long-read sequencing technologies, long reads are becoming more accurate, much longer, and more affordable [9, 10]. Accordingly, numerous long-read whole-genome de novo assemblers [11–19] have been developed and are widely applied to small-scale [20–22] and consortium projects [3, 4, 23].

Despite these advancements, it is challenging to achieve high-quality assembly, even for long reads. The algorithms of assemblers differ greatly, and each assembler typically includes a wide range of parameters. Moreover, the input data may originate from individual or multiple platforms with varying read lengths. For long-read assemblers, the input may include hybrid reads, long noisy reads (PacBio raw CLR or Nanopore), HiFi reads, reads from trio samples, and other types. Additional complexity due to ploidy, genetic diversity, heterozygosity, repetitive sequences, and sequencing depth of sequenced genomes make de novo assembly even more challenging.

De novo assembly quality assessment is therefore essential both for users to obtain optimal assembly results and for developers to improve assembly algorithms. In the short-read era, Assemblathon [24, 25] guided best practices for de novo assembly. However, there are limited toolsets that can evaluate long-read assemblies. QUAST-LG [25, 26], an extension of QUAST [27], is able to evaluate large genome assemblies. It accepts sequencing data from multiple platforms and can generate reports with rich assembly metrics as well as plots. However, QUAST-LG relies heavily on existing reference genomes, which limits its application in species without a reference genome or for samples that differ substantially from reference genomes. In addition, the misassembly evaluation of QUAST-LG is easily affected by the presence of genetic variants. Although it accepts raw reads as input, only Illumina data will be used to call structural variations (SVs) with GRIDSS [28], while long reads can only be used to report simple read statistics. Even if short reads are provided, due to the insufficiency of detecting SVs from short reads [3], it is challenging to evaluate assembly errors.

Merqury [29], inspired by KAT [30], is a reference-free toolkit for evaluating assembly quality (QV), completeness, and phasing based on the *k*-mer spectra. By comparing *k*-mers in assemblies to raw reads, Merqury can estimate base-level accuracy and completeness. Nevertheless, Merqury requires high-accuracy reads as input, such as Illumina data, which limits its application on long-read-only assembly results. While it provides base-level error estimates, Merqury cannot explicitly validate structural errors.

BUSCO [31] is a rapid and accurate method for assessing genome assembly and annotation completeness based on evolutionary ortholog genes. However, BUSCO evaluates conserved genomic regions and is not informative on the most divergent sequences in the genome.

Assembly polishing following de novo assembly is a typically used approach for improving assembly quality for downstream genomic analysis. Most current polishing algorithms correct assembly errors based on read-to-assembly alignment, as used in Racon [32], Pilon [33], GCpp [34], and CONSENT [35]. Other algorithms use *k*-mer-based approaches, such as POLCA [36] and ntEdit [37]. Nanopolish [20] and Medaka [38] polishing methods have been designed particularly for Oxford Nanopore data. Most polishing methods target small-scale errors for correction, while polishing performance on a larger scale remains unknown due to a lack of efficient evaluation methods. Another limitation is that these polishing methods often require excessive computational resources for large genomes, such as mammal genomes.

**Fig. 1:**
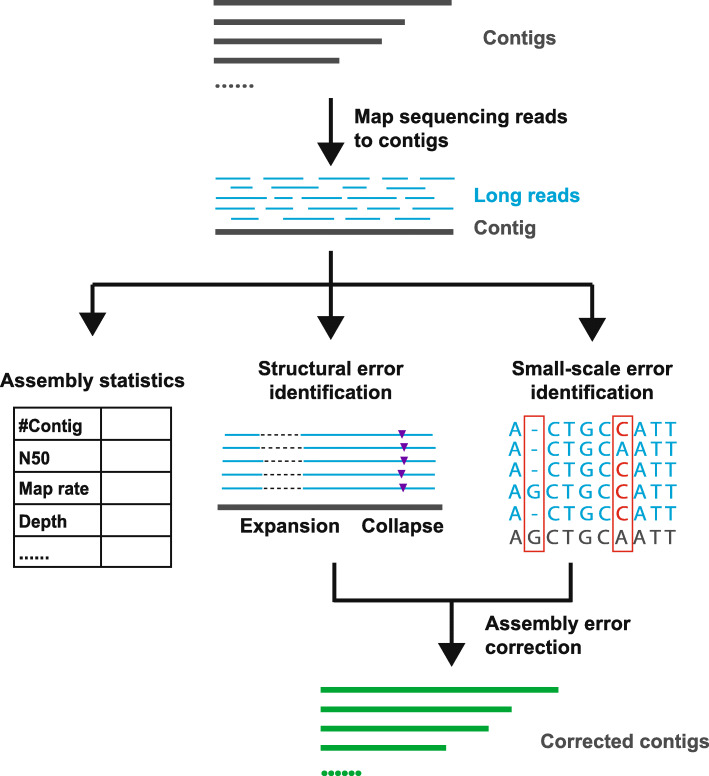
Inspector workflow for evaluating of de novo assembly results. By mapping the long reads to the contigs, besides basic statistic assembly evaluation metrics, Inspector calculates and reports precise structural errors and small-scale errors. The identified errors can also be corrected by Inspector to generate more accurate contigs

We have developed Inspector [39] to comprehensively evaluate assembly quality and identify assembly errors in haploid and diploid genomes. Instead of relying on reference genomes, Inspector evaluates assemblies with only third-generation sequencing reads, which are the most faithful representations of target genomes. By aligning sequencing reads to the contigs with minimap2 [40], Inspector generates read-to-contig alignment and performs downstream assembly evaluation (Fig. 1). Statistical analysis is initially performed to assess contig continuity and completeness. Structural assembly errors and small-scale assembly errors are identified from read-to-contig alignment and distinguished from genetic variants based on the ratio of error-containing reads. Inspector includes a targeted error correction module that addresses identified errors to improve local assembly quality. The output of Inspector includes an evaluation summary report, list of structural errors, list of small-scale errors, read alignment file, and evaluation plots.

## Results

### Small-scale assembly errors and structural assembly errors

We have classified assembly errors into two groups, small-scale errors (< 50 bp) and structural errors (≥50 bp). Small-scale errors consist of three types: base substitution, small collapse, and small expansion (Additional file 1: Fig. S1). Small-scale errors can be directly inferred from the pileup results of read alignments and filtered based on the number of error-supporting reads (“Methods”). We also have defined four types of structural assembly errors: expansion, collapse, haplotype switch, and inversion (Additional file 1: Fig. S2). Collapse and expansion are reported when part of the target genome sequence is incorrectly collapsed or expanded in the assembly. For example, collapse and expansion can occur within repetitive regions, as the presence of repeat units often forms bifurcated paths on assembly graphs, which are difficult to resolve. Haplotype switches occur at heterozygous SV breakpoints, when two haplotypes are different. The assembler fails to reconstruct either haplotype but instead generates a sequence somewhat between the two haplotypes. In these cases, reads from one haplotype will suggest a “Collapse,” and reads from the other haplotype will suggest an “Expansion.” Inversions occur when a section of the target genome sequence is inverted in the assembly.

### Benchmark with simulation

To benchmark the accuracy of assembly error detection of assembly evaluators, we compared Inspector with two other long-read assembly evaluators, Merqury and QUAST-LG, on the simulation dataset. We simulated a human genome from the reference genome (GRCh38) and introduced 1,000,000 single nucleotide and 20,000 structural variants. The SV size spectrum follows a geometric distribution similar to a real human genome [1] (Additional file 1: Fig. S3). A total of 2000 structural errors and approximately 580,000 small-scale errors (base substitutions and 1 bp indels) were randomly embedded into the simulated assembly (Additional file 2: Table S1). PacBio CLR-like reads and HiFi-like reads were simulated by PBSIM [41] and provided for Inspector to identify assembly errors. The reported assembly errors and problematic *k*-mers were compared to the ground truth to assess the accuracy of error identification for each evaluator.

Under the default settings, Inspector achieved the highest accuracy (F1 score) for assembly error detection in both haploid and diploid genomes (Table 1). For structural errors, Inspector correctly identified over 95% of simulated errors with both PacBio CLR and HiFi data. It achieved slightly better accuracy when working with HiFi data than CLR, as HiFi reads contain fewer sequencing errors. The precision was over 98% in both haploid and diploid simulations, although the number of SVs was approximately ten times greater than the true structural errors. For small-scale errors, the accuracy of Inspector was over 99% when working with HiFi data. The recall for small-scale error detection was lower (~ 86%) for CLR data, due to the lower signal-to-noise ratio caused by a higher sequencing error rate. In particular, the recall for base-substitution error was higher than for small collapse or expansion, as the latter two subtypes are more susceptible to sequencing errors (Additional file 1: Fig. S4). Most false-negative small-scale errors exhibited a lower ratio of error-supporting reads and were filtered out by Inspector for failing to reject the null hypothesis of the binomial test. The precision of small-scale error detection was over 96% for both PacBio CLR and HiFi data, benefiting from the stringent filter implemented in Inspector. Merqury identified ~ 71% of the assembly errors with a precision of ~ 91.6% on both CLR and HiFi data. Merqury failed to detect more small collapses than base substitution and small expansions, and over 70% of Merqury-missed small-scale errors were located in repeat regions (Additional file 1: Fig. S5). QUAST-LG had much lower recall and precision than Inspector and Merqury, as some misassemblies were indeed caused by SVs (18% in haploid and 36% in diploid). In both haploid and diploid simulated assemblies, Inspector detected the structural assembly errors and small-scale errors with the highest accuracy among the three evaluators.

**Table 1:** Assembly error identification accuracy in simulated assembly

	Haploid				Diploid		
	Recall/%	Precision/%	F1 score/%		Recall/%	Precision/%	F1 score/%
Inspector structural – CLR	96.76	100.0	98.35		95.98	98.48	97.21
Inspector structural – HiFi	97.64	100.0	98.80		97.61	98.87	98.23
Inspector small-scale – CLR	86.84	99.53	92.75		86.60	96.99	91.50
Inspector small-scale – HiFi	98.99	99.65	99.32		98.91	99.62	99.26
Merqury	71.01	91.66	80.03		70.92	91.63	79.95
QUAST-LG	5.73	5.96	5.84		7.08	8.48	7.72

### Human genome assembly evaluation

We next performed whole-genome de novo assembly on a real human genome and evaluated the assembly results. We used an Ashkenazi Jewish sample, HG002, from Genome in a Bottle (GIAB) for the analysis. This sample has been sequenced by multiple platforms, including PacBio CLR, PacBio HiFi, Oxford Nanopore, and Illumina. There are experimentally or multiple-platform validated SNP/indel callset and SV callset at high-confidence regions publicly available for this sample [42–44], which enables the validation of identified assembly errors. We tested five state-of-the-art assemblers, Canu [14], Flye [15], wtdbg2 [16], hifiasm [19], and Shasta [17], on the PacBio CLR (~70×), HiFi (~55×), and Nanopore (~60×) dataset, if applicable. Besides Inspector, we have applied Merqury and QUAST-LG to evaluate and compare the assembly results (Table 2).

**Table 2:** Evaluation summary of HG002 assemblies

Assembly			Contig continuity					Assembly error				QUAST-LG			Merqury		Reference-based mode		
			# Contig	Total	Max	N50		Structural	Small-scale	QV		Misassembly	MM		QV		NA50	MR (%)	Coverage (%)
**CLR**																			
	Canu		4751	2.91	72.0	7.2		103	39.82	43.63		8341	18.84		38.51		1.32	99.15	89.41
	Flye		2168	2.82	66.6	12.0		192	30.88	43.38		4005	16.46		38.71		1.47	99.36	88.67
	Wtdbg2		2947	2.77	48.5	7.0		158	430.00	33.46		8943	29.13		29.42		0.43	97.77	86.17
**HiFi**																			
	Canu		1376	3.37	192.2	65.3		5	1.90	54.85		47672	29.17		46.57		2.20	95.95	91.71
	Flye		2379	2.96	136.6	35.1		256	20.74	43.69		14478	17.34		48.08		2.28	97.82	90.36
	wtdbg2		1652	2.76	74.8	16.3		251	83.06	39.42		4124	14.65		42.66		1.56	99.38	86.77
	hifiasm		559	3.07	199.4	111.1		18	3.62	53.62		31143	21.47		45.88		2.53	97.37	92.03
**Nanopore**																			
	Canu		745	2.90	101.3	33.1		1432	3845.99	24.05		14926	100.03		22.94		0.27	98.27	88.46
	Flye		584	2.87	109.9	51.7		481	316.46	34.30		7688	33.94		30.46		1.48	99.32	89.80
	wtdbg2		7959	2.97	54.2	8.2		2226	2116.76	24.91		23159	65.88		24.49		0.30	93.79	84.91
	Shasta		1258	2.80	129.3	23.3		2527	2554.72	25.74		9063	70.15		24.76		0.31	99.16	87.71

The unit of Max, N50, and NA50 is Mbp. The unit of Total is Gbp. The unit of small-scale and MM is per Mbp. Misassembly of QUAST-LG includes both extensive and local misassembly. Mismatch of QUAST-LG includes both mismatches and indels

*Total* total number of bases, *Max* length of the longest contig, *MM* number of mismatches, *MR* mapping ratio of assembled contigs

Inspector first estimated assembly continuity. Most assemblies contained a total of 2.7–3.0 giga base pairs, close to the reference genome, suggesting that these assemblers can reconstruct the overall structure of the target genome using long reads. Based on the maximal contig length and the N50, the sequence length of the shortest contig at 50% of the total contig lengths, Flye achieved the best continuity in the PacBio CLR and Nanopore datasets, while hifiasm outperformed the other assemblers in the HiFi dataset. Inspector then aligned the sequenced reads to contigs and identified assembly errors from read-to-contig alignments. Canu introduced the fewest structural errors as well as small-scale errors in CLR and HiFi assemblies. Hifiasm achieved results similar to Canu. Nanopore assemblies contained much more structural errors and small-scale errors than CLR and HiFi assemblies. This was likely due to the higher error rate of the Nanopore sequencing data. Flye generated the most accurate assembly among the four assemblers with Nanopore data. Note that the assemblers were tested using their default or recommended parameters. Optimized de novo assembly results by fine-tuning the parameters of individual assemblers may render different evaluation results.

For an overall evaluation of assembly quality, we introduce the Quality Value (QV) score. QV score is calculated based on the identified structural and small-scale errors scaled by the total base pairs of the assemblies (“Methods”). In general, PacBio HiFi assemblies demonstrated higher QV scores than CLR and Nanopore assemblies. Canu achieved the highest QV score in PacBio CLR and HiFi datasets, and Flye outperformed other assemblers in Nanopore dataset. We also evaluated all assemblies using Merqury. QV scores calculated by Merqury highly correlated with Inspector’s results (Additional file 1: Fig. S6). QUAST-LG was also used to evaluate the assemblies. As the SVs were not excluded from the misassembly list, the total number of misassemblies was much larger than Inspector’s result in all assemblies.

When the reference genome is available, Inspector can also assess the assembly synteny by aligning contigs to the reference genome. Based on the contig-to-reference alignment, Inspector computes NA50 (N50 calculated on the basis of aligned blocks instead of contig lengths), contig mapping ratio, and reference genome coverage for each assembly, reflecting the completeness of the assembly. Inspector also generates N1-N100 plots (Additional file 1: Fig. S7) and Dotplot (Additional file 1: Fig. S8) to reflect the consistency between the assembly and reference genome

NA50 and reference genome coverage in HiFi assemblies were larger than the CLR and Nanopore assemblies, which suggests that HiFi assemblies were more complete and more consistent with the reference genome. Because the reference genome is different from the evaluated genome, these statistics may be slightly affected by genetic variants. Overall, we found that HiFi assemblies were more accurate and complete than CLR and Nanopore assemblies, suggesting that better assembly results can be achieved from long and accurate sequences.

### Distinguish assembly errors from genetic variants

Inspector distinguishes assembly errors from genetic variants mainly from the number of reads that support the error. We identify them as “error-supporting” reads. The expected ratio of error-supporting reads is higher for assembly errors than genetic variants (Additional file 1: Fig. S9, S10). For small-scale errors, Inspector counts the number of reads supporting errors and contigs, and then performs binomial test to select assembly errors with significant *p* values depending on the input data (“Methods”). For structural errors, a stringent filter of assembly errors is designed to sift out SVs based on the ratio of error-supporting reads and other features such as read mapping quality. We have defined the false discovery rate (FDR) of assembly error in HG002 as the errors that are actually genetic variants. We compared the identified assembly errors to the high-confidence variant callsets and computed the FDRs in each assembly. Inspector efficiently distinguished small-scale and structural (collapse and expansion) assembly errors from genetic variants, with an average FDR of 2.88% and 1.15%, respectively (Table 3). The FDR for Merqury and QUAST-LG were both higher than for Inspector. We also evaluated accuracy for haplotype switches and validated that over 90% of the reported events occurred near heterozygous SV breakpoints (Additional file 2: Table S2).

**Table 3:** False discovery rate of assembly errors in HG002 assemblies

		Inspector			Merqury	QUAST-LG
		Small-scale	Structural			
**CLR**	Canu	3.57	–^a^		14.36	35.23
	Flye	5.77	0.00		21.93	51.65
	wtdbg2	0.94	0.00		15.33	38.37
**HiFi**	Canu	6.21	–^a^		3.61	38.96
	Flye	0.41	0.00		56.13	52.64
	wtdbg2	0.90	2.38		72.64	64.23
	hifiasm	8.85	0.00		9.99	51.63
**Nanopore**	Canu	1.01	0.00		3.89	23.16
	Flye	1.28	7.69		5.22	52.39
	wtdbg2	0.72	0.00		6.37	12.32
	Shasta	1.96	0.32		5.15	46.68
**Mean**		2.88	1.15		19.51	42.48

^a^Assemblies with no structural error located in the benchmark regions of HG002 are marked with “–”

**Fig. 2:**
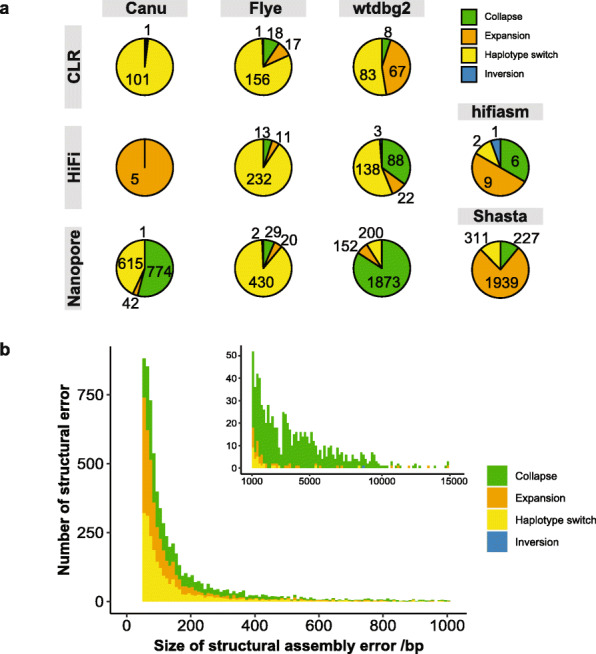
Characterization of structural assembly errors in HG002 assemblies. **a** Pie charts showing the proportion of four types of structural errors identified in Canu, Flye, wtdbg2, hifiasm, and Shasta assemblies with CLR, HiFi, and Nanopore datasets, respectively. The number of assembly error is also marked on each sector. **b** Size distribution of identified structural assembly errors in all HG002 assemblies

We further characterized the structural errors identified from these assemblies (Fig. 2a). The error patterns varied among the assemblers and among different data types. For example, Flye consistently showed a predominance of haplotype switches, suggesting a possible systematic error when assembling the heterozygous regions. In addition, Canu and wtdbg2 showed more collapses in Nanopore assemblies than PacBio CLR and HiFi assemblies. This may be due to a higher deletion error rate in Nanopore data, in contrast to a higher insertion error in PacBio data. In general, structural errors were dominated by relatively small errors, with 84.8% of structural errors shorter than 500 bp (Fig. 2b). Collapses accounted for 88.9% of structural errors that were larger than 1 kbp. Inversion errors were much rarer than the other three types and were usually large in size (493 kbp on average). The error pattern of small-scale errors also varied among assemblers but showed more consistency within the same data type (Additional file 1: Fig. S11).

To assess the effect of sequencing depth on Inspector’s evaluation performance, we merged three HiFi datasets from GIAB and downsampled to a series of depths ranging from 10× to 100×. We evaluated the same assembly with these downsampled HiFi datasets. The number of assembly errors reported by Inspector was stabilized when the sequencing depth was higher than 30× (Additional file 1: Fig. S12), which suggests that the sequencing depth has minor effect on Inspector’s error detection, and a 30× dataset is sufficient for accurate assembly evaluation with Inspector.

### Assembly errors are enriched in repetitive regions

**Fig. 3:**
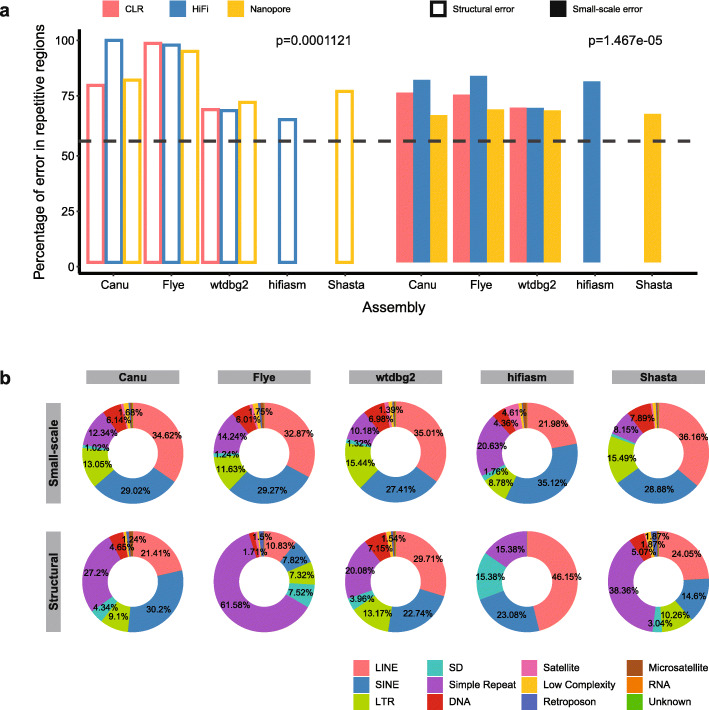
Enrichment of assembly errors in repetitive regions. **a** Proportion of assembly errors located in repetitive regions in each assembly. Dashed line indicates fraction of human reference genome annotated as repeats. *P* values were calculated by one-sample *t*-test to compare the proportion of assembly errors with the baseline. **b** Repeat annotation of structural and small-scale errors for five assemblers

Inspector reports precise locations of structural and small-scale errors, which allows us to further annotate assembly errors from each assembly result. We projected the coordinates of identified assembly errors to the reference genome and annotated these assembly errors (“Methods”). To ensure accurate repeat analysis, we used HiFi data to identify small-scale errors in all assemblies. We found that both structural errors and small-scale errors were enriched in the repetitive sequences (Fig. 3a). Given that approximately 55% [45] of the human genome is annotated as repetitive sequences [45], we observed a significantly higher proportion of structural (82.09%) and small-scale (73.61%) errors located in repetitive regions, suggesting that repeats remain challenging for long-read de novo assembly. We further examined the seven types of repetitive sequences that each account for more than 1% of the reference genome (Additional file 1: Fig. S13). We found that both structural and small-scale errors were enriched in simple repeats. The average percentage of structural errors located in simple repeats was 45.9%, which was a ten-fold enrichment compared to the genome baseline. Small-scale errors were also enriched in LINE, SINE, LTR, and DNA repeat elements for these assemblies as a whole. We observed an overall lower percentage of errors located in the segmental duplication and satellite regions, although some assemblies showed a higher-than-expected assembly error rate.

We next characterized the repeat-associated assembly errors for the five tested assemblers. The composition of different types of repeats was relatively consistent for small-scale errors among the five assemblers tested (Fig. 3b), with majority of errors located in LINE, SINE, simple repeat, and LTR regions. When separating assemblies from three different data types, we observed consistent patterns in CLR assemblers and Nanopore assemblies (Additional file 1: Fig. S14). In the four HiFi assemblies, there was strong enrichment of simple repeats in the Flye assembly, suggesting that Flye may have worse base accuracy when resolving simple repeat regions than other genomic regions. For the structural errors, both Flye and Shasta (merely applicable to Nanopore data) demonstrated strong enrichment in simple repeats than the other three assemblers (Fig. 3b). This enrichment is consistent in PacBio CLR, HiFi, and Nanopore assemblies for Flye (Additional file 1: Fig. S15). Taken together, Inspector revealed the enrichment of assembly errors in repetitive regions and distinct repeat enrichment patterns of different assemblers, which provides guidance for further development and improvement of assemblers.

### Assembly error correction

**Fig. 4:**
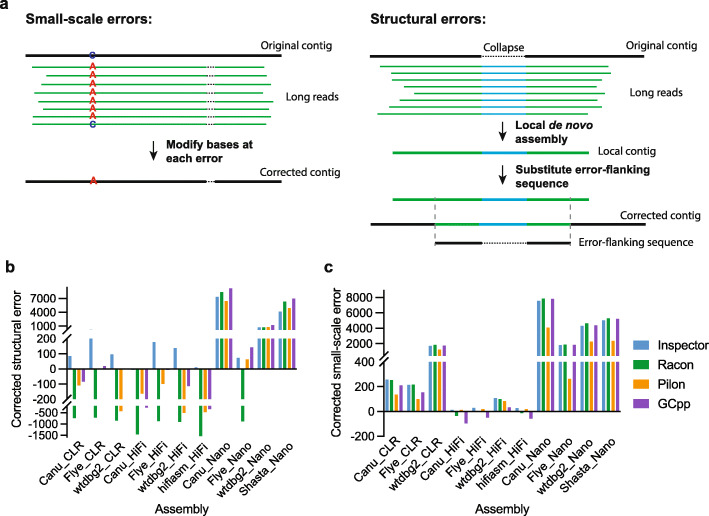
Improved assembly accuracy after error correction. **a** Methods of assembly error correction for small-scale and structural errors. **b, c** Number of corrected structural (**b**) and small-scale errors (**c**) in HG002 assembly. Negative values indicate more assembly errors after the polishing process

Equipped with the coordinates of assembly errors, Inspector includes an error correction module for improving assembly quality, which facilitates downstream analysis. The error correction module eliminates highly confident assembly errors (Fig. 4a). Small-scale errors are corrected by replacing misassembled bases at reported locations. Structural errors are corrected by performing local de novo assembly around each error (“Methods”). Because the local assembly utilizes sequencing reads from only this locus (and from only one haplotype for haplotype switches), the newly generated contig can reconstruct the target genome more accurately and can therefore fix structural assembly errors.

We evaluated genome polishing performance of the Inspector error correction module and six state-of-the-art alignment-based polishing methods, including Racon, Pilon, GCpp, Medaka, Nanopolish, and CONSENT on HG002 assemblies from Canu, Flye, wtdbg2, hifiasm, and Shasta. We used one HiFi dataset of HG002 for polishing and used another HiFi dataset to evaluate the original and polished assemblies to avoid bias (“Methods”). After polishing with HiFi reads, Inspector corrected most structural errors among four tested polishing tools in the CLR and HiFi assemblies, while GCpp corrected most structural errors in the Nanopore assemblies (Fig. 4b). Nevertheless, in CLR and HiFi assemblies, there were more structural errors after polishing with Racon, Pilon, and GCpp, suggesting that these polishing methods can correct structural errors in lower-quality assemblies but may introduce more structural errors in relatively accurate assemblies. For small-scale errors, Inspector, Racon, and GCpp achieved higher error correction rates than Pilon in most assemblies (Fig. 4c). GCpp introduced more small-scale errors in the HiFi assemblies. Based on the increased QV score after polishing, Inspector outperformed other polishing methods in CLR and HiFi assemblies, while Racon achieved the best QV score improvement in Nanopore assemblies (Additional file 1: Fig. S16a). Estimation of QV score with Merqury also supported that Inspector and Racon achieved the highest assembly quality among the tested polishing methods (Additional file 1: Fig. S16b).

When polishing the assemblies with CLR and Nanopore reads, Racon, CONSENT, and Medaka introduced new structural errors after polishing the CLR and HiFi assemblies (Additional file 1: Fig. S17). The number of small-scale errors in CLR and HiFi assemblies was also increased after polishing with noisy reads, especially with Nanopore reads. Inspector and Pilon reduced assembly errors or introduced the fewest errors when given noisy reads as inputs for polishing. Compared with polishing using CLR and Nanopore reads, Inspector achieved the highest error correction rate using HiFi reads for both small-scale errors (Additional file 2: Table S3) and structural errors (Additional file 2: Table S4), owing to the highest base accuracy of the HiFi dataset.

We also evaluated short-read polishing on the HG002 assemblies. Although the small-scale errors were reduced in all assemblies (Additional file 1: Fig. S18a), the number of structural errors increased in most assemblies after short-read polishing with Racon or Pilon (Additional file 1: Fig. S18b). QV scores estimated by Inspector and Merqury were both increased in CLR and Nanopore assemblies but showed minor or no improvement in HiFi assemblies (Additional file 1: Fig. S18c), suggesting that additional high-accuracy short-read datasets can only improve the quality of assemblies generated from noisy long reads.

In addition to the human genome, we also tested the Inspector error correction module on the genome of Anna’s hummingbird (*Calypte anna*) [46]. We performed whole-genome assembly with Canu, Flye, and wtdbg2 and corrected identified assembly errors using Inspector. The number of structural errors and small-scale errors both dropped after Inspector error correction, with increased QV scores for all assemblies (Additional file 1: Fig. S19). We also compared the original and Inspector-corrected assemblies to the curated genome to validate that the structural errors in the original assemblies were accurately corrected by Inspector (Additional file 1: Fig. S20). Taken together, the error correction module of Inspector can improve assembly quality by correcting both structural and small-scale errors and can achieve better error correction efficiency than other polishing methods in more accurate assemblies.

### Runtime and memory usage

Inspector and other assembly evaluation and polishing methods were tested on Intel Xeon E5-2680 v3 CPUs with 2.5 GHz. It took 13.6 h to evaluate a human genome assembly (Canu assembly of HG002) using 50× PacBio HiFi dataset with peak memory of 35 GB (Additional file 2: Table S5). The error correction of this assembly took 26 min with peak memory of 17GB (Additional file 2: Table S6).

## Discussion

We have developed a reference-free long-read de novo genome assembly evaluator, Inspector, which reports exact locations, sizes, and types of assembly errors without being affected by genetic variants. In addition, Inspector improves assembly results by correcting discovered errors. These features are unique to Inspector and have not been achieved by other available assembly evaluators. We also performed detailed error analysis on different assemblers applied to different datasets. As expected, errors appear predominantly in repetitive regions. However, not all types of repeats are enriched with assembly errors. This information is important for the investigation of systematic defects in assembler algorithms. Therefore, Inspector can provide guidance for users and developers on achieving optimal assembly results.

Inspector implements multi-thread processing for read alignment, assembly error identification, and assembly error correction. For identification and correction of assembly errors, Inspector processes one contig per thread, which largely reduces runtime and memory usage. The read alignment by minimap2 is the most time-consuming step in Inspector evaluation (accounting for approximately 70% of total runtime). Therefore, the runtime of Inspector largely depends on the sequencing depth of the input dataset. The total runtime for Inspector is longer than for Merqury and QUAST, but it requires much less memory (Additional file 2: Table S5). For assembly error correction, the runtime of Inspector depends on the number of structural errors present in the assembly, as Inspector performs local assembly for each error. Inspector used shorter computing time and less memory than Racon, Pilon, GCpp, and Medaka (Additional file 2: Table S6), benefiting from known the error positions from previous evaluation results. Nanopolish and CONSENT both required excessive computing resources for whole-genome polishing (requiring over 10 days for polishing one human genome) and thus were tested on only one contig.

Detecting assembly errors from read-to-contig alignment is a challenging problem similar to detecting genetic variants from read-to-reference alignment. Identification of small-scale error is extremely challenging with error-prone reads. The abundance of sequencing errors not only introduces ambiguity in read alignment but also reduces signal strength during error detection. To ensure high precision of assembly error detection, Inspector applies a stringent filter to exclude heterozygous variants, which will lead to a lower recall for small-scale errors in the CLR data, as shown in Table 1. In the real PacBio datasets, the HiFi data also reported lower QV score and more assembly errors, especially small-scale errors, than the CLR data. This is because the accurate HiFi reads are more sensitive for detecting errors. Advanced algorithms for better characterization of small-scale variants can improve the sensitivity of error detection from noisy sequencing data. When available, we will include this enhancement in future Inspector releases.

In this work, we have described our methods for benchmarking and analysis of human and Anna’s hummingbird genomes. Inspector can also be applied to other species with monoploid or diploid genomes. The principles of structural error identification and binomial testing for small-scale errors are both designed with the assumption that a genome is diploid. These principles are also applicable to a haploid genome, which can be considered as an extreme case of a diploid genome with only homozygous bases. Evaluation for species with higher ploidy levels may not be as accurate under the current version. With further development, we plan to expand the application of Inspector to species with polyploid genome in future versions.

## Conclusions

This paper presents a reference-free evaluation method for de novo assembly. Inspector can report the precise locations and sizes for structural and small-scale assembly errors and distinguish true assembly errors from genetic variants. With its error correction module, Inspector can improve the assembly quality by correcting the identified assembly errors. These functions exceed those achieved by existing assembly evaluators. Inspector is an accurate assembly evaluator, which can facilitate future improvement of de novo assembly quality.

## Methods

### Overview of Inspector

Inspector is a tool for evaluating long-read de novo assembly results. As shown in Fig. 1, inspector consists of the following main functions: (1) standard assembly metrics; (2) structural error identification; (3) small-scale error identification; and (4) assembly error correction. Inspector also introduces a Quality Value (QV) to estimate the overall assembly quality. Given a reference genome, Inspector can assess synteny by aligning contigs to the reference genome. The detailed methods and implementation are described below.

### Contig continuity and read alignment

Inspector first calculates standard assembly statistical metrics and then evaluates contig continuity based on the lengths of all contigs. Standard statistical metrics include number of contigs, total bases in the assembly, longest and second longest contig lengths, and N50, which reflect continuity of assembly results.

The statistics of read-to-contig alignments are also calculated to assess assembly quality, including read mapping rate, read splitting rate, and average alignment depth. Read mapping rate indicates the proportion of reads that can be aligned to assembled contigs. A higher read mapping rate suggests better completeness of the assembly, while a lower mapping rate suggests that parts of the genome have not been reconstructed in the assembly. The read splitting rate is the proportion of aligned reads that have split alignments. A low read splitting rate indicates better consistency between reads and assemblies and fewer large assembly errors. In contrast, a high splitting rate suggests that there are more assembly errors which have caused the divergence between reads and assembled contigs. The average alignment depth is calculated as total length of aligned reads divided by total contig length. For good assembly, average alignment depth should be similar to sequencing depth of input reads.

### Structural assembly errors

Inspector detects structural assembly errors (≥ 50 bp) based on disagreement between reads and assembled contigs. The first step is to scan all read alignments for raw error signals of expansion (gap in read alignment), collapse (extra sequence in read), and inversion (inverted read alignment). Density-based clustering is then performed independently for each type of structural error. Instead of setting a fixed window size for clustering raw signals, Inspector’s density-based clustering utilizes adjustable window size to tolerate larger shifts of raw signal positions within repetitive regions while keeping tight window size for clear genomic regions. Expansions and collapses are merged to identify haplotype switches, in which expansions overlap with collapses. To remove noise caused by sequencing errors or incorrect read alignments, Inspector filters out candidates with numbers of supporting reads below a threshold value (three by default).

To remove false-positive candidates caused by genetic variants, Inspector includes a filter based on the ratio of error-supporting read, local coverage, and read mapping quality. The ratio of error-supporting read is the fundamental criterion and computed with the number of error-supporting reads divided by the local coverage. As shown in Additional file 1: Fig. S9, read alignments at homozygous variants do not show inconsistency with the contig, as both haplotypes are the same as the contig sequence. Heterozygous variant regions show an alternative allele in about 50% of reads (from one haplotype). However, at true assembly error regions, both haplotypes are different from the contig, including the haplotype switch, leading to a theoretical ratio of about 100% for error-supporting reads. The ratio of error-supporting read for assembly errors can be lower than 100% in practice due to sequencing errors or inaccurate read alignments but are still higher than heterozygous variants, as shown in Additional file 1: Fig. S10. The filter also discards candidates with extremely high coverage or poor average read mapping quality to ensure the reported assembly errors are confident. By default, Inspector reports coordinates on contigs for all assembly errors in BED format, which can be easily loaded to visualization tools such as IGV [47].

### Small-scale assembly errors

Inspector identifies small-scale assembly errors (< 50 bp) to estimate the base accuracy of an assembly. Samtools [48] is used to generate pileup information for each contig based on read-to-contig alignments. Inspector then scans pileup results for candidate small-scale errors in regions that are enriched with mismatches or indels. All bases with less than 20% of reads supporting a small-scale error were excluded to remove most noise caused by sequencing errors. Similar to structural errors, a true small-scale error is expected to be supported by reads from both haplotypes (100% of reads), while mismatches or indels caused by heterozygous variants are supported by only one haplotype (50% of reads). For a given position on the assembly, each aligned read is treated as an independent experiment, containing either the same or a different base (or indel) with the base in the contig. All bases in the reads at this position follow a binomial distribution, with *n* being the number of reads and *p* being the probability that the base is a different base from the contig. Inspector performs a one-tailed binomial test for each candidate position to distinguish small-scale errors from genetic variants. The null hypothesis of the binomial test is that the probability of a read that contains a different base against the contig is 0.5 (genetic variant at this location), and the alternative hypothesis is that the probability is higher than 0.5 (small-scale error at this location). A significant *p* value from the binomial test would reject the null hypothesis and support that there is a small-scale error at the tested position. The *p* value of binomial test is computed as:

$$ p\_\mathrm{value}=\sum \limits_{i={n}_{\mathrm{supp}}}^{n_{\mathrm{reads}}}\mathrm{Binomial}\left(i|p=0.5,n={n}_{\mathrm{reads}}\right) $$

where *n*_reads_ is the total number of reads aligned to this position and *n*_supp_ is the number of reads supporting the mismatch/indel. The probability of a read to support an error used in binomial test is set to 0.5 for high-accuracy HiFi data, and set to 0.4 for low-accuracy data (CLR and Nanopore), considering the sequencing error rate of 15–20%. Candidates with significant *p* values (< 0.01 for HiFi and < 0.05 for CLR and Nanopore data) are reported as small-scale errors. Similar to structural errors, small-scale errors are also reported in BED format.

### Assembly quality estimation

Structural and small-scale assembly errors are used to estimate the overall accuracy of an assembly result. Given a list of structural errors and small-scale errors of the assembly, the total bases of assembly error, *N*_*Err*_, can be calculated as:

$$ {N}_{Err}={N}_{Exp}+{N}_{Col}+{N}_{Her}+{N}_{Small}+{n}_{Inv} $$

where *N*_*Exp*_ , *N*_*Col*_, *N*_*Her*_, and *N*_*Small*_ are the total bases affected by expansions, collapses, haplotype switches, and small-scale errors, while *n*_*Inv*_ is the total number of inversion errors. Since the number of total bases in an assembly, *N*_*asm*_, is usually very large, *N*_*Err*_ can be considered as the expectation of incorrect bases. Thus, the estimated error rate, *E*, can be defined as:

$$ E=\frac{N_{\mathrm{Err}}}{N_{\mathrm{asm}}}=\frac{N_{\mathrm{Exp}}+{N}_{\mathrm{Col}}+{N}_{\mathrm{Her}}+{N}_{\mathrm{Small}}+{n}_{\mathrm{Inv}}}{N_{\mathrm{asm}}} $$

The Phred quality score is computed as *QV* =  − 10*log*_10_*E*.

### Assembly error correction

Inspector includes an error correction module to address identified structural and small-scale assembly errors. For small-scale errors, Inspector substitutes problematic bases with bases supported by the majority of reads. For structural assembly errors, Inspector collects the error-supporting reads and performs a local de novo assembly with Flye (v2.8.3) [15] for each assembly error. In particular, for haplotype switches, Inspector only collects reads from one haplotype to perform the local assembly. For each structural error, the local assembly uses the reads from the region around the error and from the same haplotype, which simplifies the assembly process and can therefore generate a more accurate contig than whole-genome de novo assembly. For structural errors located within repetitive regions, Inspector collects reads only from the current repeat unit without interference from other repeat units, increasing the accuracy of local assembly at repetitive regions. Inspector aligns the new contigs from local assemblies to the original contigs and substitutes the sequences flanking each error with new sequences from the local assembly results.

### Reference-based mode

To assess the synteny of an assembly with a known reference genome, Inspector includes a reference-based module to evaluate assembly quality. The module aligns contigs to the reference genome with minimap2 [40] preset parameter “-x asm5.” Statistics for contig-to-reference alignment are calculated, including contig alignment NA50, contig mapping rate, and reference genome coverage. A Dotplot is generated based on contigs and reference alignment results. In addition, structural errors and small-scale errors are detected. Inspector reports coordinates on the reference genome and on the contig for all assembly errors. Note that assembly errors detected from contig-to-reference alignment also include genetic variants of the sequenced genome (including SVs, SNPs, and indels) and substitutions.

### Simulation benchmark

To benchmark the evaluation accuracy of Inspector, testing used a simulated human whole-genome assembly containing both structural and small-scale assembly errors. A total of 1,000,000 SNPs and 20,000 SVs (deletions and insertions) were introduced into autosomes and X chromosome of human reference genome hg38. In total, 67% of all variants were randomly assigned as heterozygotes and 33% as homozygotes. PBSIM [41] was used to simulate 50X PacBio CLR-like and HiFi-like reads with options “--data-type CLR --model_qc model_qc_clr --length-mean 15000 --length-sd 3000 --accuracy-mean 0.85” and “--data-type CCS --model_qc model_qc_ccs --length-mean 15000 --length-sd 3000 --accuracy-mean 1.00,” respectively. The mean base accuracy was 0.85 for CLR-like reads and 0.98 for HiFi-like reads according to the log file from PBSIM. Assembled contigs were simulated by splitting the simulated human genome at “*N*” bases. Small fragments shorter than 10,000 bp were excluded. A total of 2000 structural errors (900 expansions, 900 collapses, 190 haplotype switches, and 10 inversions) and about 580,000 small-scale errors (50% base substitution, 25% 1-bp expansion, and 25% 1-bp collapse) were spiked in as the ground truth. A haploid human genome was also simulated by selecting only haplotype 1 from the diploid simulation.

Inspector was applied with default settings. The reported structural and small-scale errors were compared to the ground truth to calculate recall, precision, and F1 score ($\frac{2\ast \mathrm{recall}\ast \mathrm{precision}}{\mathrm{recall}+\mathrm{precision}}$). Human reference genome hg38 was provided to QUAST-LG as the reference. Although the minimum length for structural errors was 50 bp in simulated assemblies, QUAST-LG can only report the coordinates of extensive misassemblies longer than 85 bp. These extensive misassemblies were compared with a subset of ground-truth structural errors that were longer than 85 bp to assess the accuracy of QUAST-LG. Since Merqury requires high-accuracy reads as input data, the simulated HiFi dataset (with sequencing error rate < 2%) was provided to Merqury to identify erroneous *k*-mers that were only present in the assembly but not in the input reads. A series of overlapping *k*-mers were merged into one single event for the benchmark.

### Whole-genome de novo assembly of HG002

Whole-genome de novo assembly was performed for HG002 with PacBio CLR, HiFi (15-20 kb), and Nanopore datasets. The expected genome size was set to 3.1G for all assemblers. Canu (v2.0) was run with options “-pacbio” for the PacBio CLR and “-pacbio-hifi” for the PacBio HiFi dataset. The Canu assembly of the Nanopore dataset was obtained from a previous publication [17]. Contigs marked with “suggestBubble = yes” were removed from evaluation. Flye (v2.8.2) was run with options “--pacbio-raw” for the CLR, “--pacbio-hifi” for the HiFi, and “--nano-raw” for the Nanopore dataset, respectively. Wtdbg2 (v2.5) was run with options “-p 17” for the CLR and Nanopore datasets, and preset “-x ccs” for the HiFi dataset. Hifiasm (v0.13) was only applied to PacBio HiFi datasets with the default settings. The Shasta assembly of Nanopore dataset was also obtained from a previous publication [17]. All assemblies were evaluated by Inspector with default settings. CLR assemblies were evaluated with the raw CLR dataset, HiFi assemblies were evaluated with the HiFi dataset (15-20 kb), and Nanopore assemblies were evaluated with the raw Nanopore dataset.

### Other assembly evaluation tools

QUAST-LG (v5.0.2), a reference-based approach, and Merqury (v1.1), a *k*-mer based approach, were also used to evaluate assemblies. For QUAST-LG, GRCh38 was provided as the reference genome. QUAST-LG was run with command:

“quast-lg.py contig.fa -o output/ -r hg38.fa -m 10000 - × 86”

The number of misassemblies included both extensive and local misassemblies, and number of mismatches included both mismatches and indels.

For Merqury, a meryl database was first generated with approximately 50× Illumina paired-end reads with *k*-mer size of 21 bp. Merqury was then run based on the Illumina meryl database to evaluate HG002 assemblies with default settings:

“meryl *k* = 21 count output read-db.meryl allread.fa”

“merqury.sh read-db.meryl contig.fa output”

The assembly-only *k*-mers were collected from Merqury’s output and the overlapping *k*-mers were merged into a single event.

### Benchmark of assembly error in HG002

The false discovery rate of assembly errors was calculated by comparing reported assembly errors to the genetic variant callset of HG002. Coordinates of assembly errors were projected to the human reference genome based on contig-to-reference alignment. Matched base pairs between contigs and the reference genome were stored in a hash table. The corresponding reference coordinate of an assembly error can be inferred from the hash table according to its assembly coordinate. Small-scale errors were compared to the small variant callset (v4.2.1) from GIAB. Since the high-confidence SV callset is only available in “benchmark regions” of HG002 [43], structural assembly errors located only in benchmark regions were compared to the SV callset to calculate FDRs.

Coordinates of misassemblies reported by QUAST-LG were extracted from filtered contig alignment. Misassemblies located within benchmark regions were compared to the SV callset for FDR assessment. Assembly-only *k*-mers from Merqury’s output were merged and projected to the reference genome. FDR was computed by comparing the locations of *k*-mers to the merged variant callset (SVs and small variants).

### Down-sampling of HG002

To evaluate the robustness of Inspector, three HiFi datasets (11 kb, 15 kb, and 15–20 kb) of HG002 were merged to generate a HiFi dataset with an ultra-high depth. It was then downsampled to a series of depths, ranging from 10× to 100×, by randomly selecting reads. Depth was determined as total number of base pairs in reads divided by the human genome size (3.1 Gbp). Inspector was applied to identify assembly errors using default settings to validate its robustness in addressing datasets of varying depth.

### Repeat annotation of assembly errors

Coordinates of assembly errors were projected to the human reference genome. Those assembly errors located in unaligned parts of the assembly cannot be projected to the reference genome and therefore were excluded from analysis. Repeat annotation of all assembly errors was performed by a custom Python script, which compared reference coordinates of assembly errors to the genomic repeat annotation downloaded from UCSC Genome Browser [49].

### Polishing of HG002 assemblies

Inspector correction and other polishing methods were tested on HG002 assemblies. The error correction module of Inspector was tested with PacBio CLR (70×), PacBio HiFi (15–20 kbp, 51×), and Nanopore (53×) datasets with default settings. The input datatype was specified for each dataset to enable accurate local assembly in the structural error correction process. Racon (v1.4.20) and Pilon (v1.24) were tested with PacBio CLR, PacBio HiFi, and Nanopore datasets with default settings. GCpp (v 2.0.2) was tested with downsampled raw subreads of PacBio HiFi dataset (70×). Medaka (v 1.4.3) polished HG002 assemblies with Nanopore datasets with the options “--model r941_min_high_g303 --batch 200 --bam_chunk 2000000.” Nanopolish (v0.13.3) was tested with Nanopore dataset using default settings. CONSENT (v2.2.2) polished HG002 assemblies with PacBio CLR datasets with options “--windowSize 50000.” Nanopolish and CONSENT were tested on only one contig (10Mbp in length) per assembly due to the excessive requirement of computational resources for whole-genome correction. The input read alignment files for Racon, Pilon, Medaka, and Nanopolish were aligned by minimap2 and sorted by Samtools sort. The read alignment files provided to GCpp were aligned by pbmm2 and sorted by Samtools. All polishing tools were tested with only one round of the polishing process. We also polished the HG002 assemblies with Illumina dataset (downsampled to 50×) to assess the improvement of assembly quality from short reads. The original and polished assemblies were evaluated using Inspector with a merged HiFi dataset (11 kbp and 15 kbp, total of 58×) and using Merqury with meryl database generated from Illumina dataset.

### Whole-genome assembly of Anna’s hummingbird sample

The PacBio CLR (~70×) data of Anna’s hummingbird (*Calypte anna*) was downloaded from the Vertebrate Genomes Project and used to for whole-genome de novo assembly with Canu, Flye, and wtdbg2 with genome size of 1.1 Gbp. Inspector was run with default settings to evaluate and correct errors for the three assemblies. The curated assembly was obtained from GenomeArk as the ground truth. The uncorrected and corrected assemblies were compared to curated assembly with Mauve [50] to visualize structural errors before and after Inspector error correction.

## Supplementary Information

**Additional file 1: Supplementary Fig. S1-S20.**

**Additional file 2: Supplementary Tables S1-S6.**

**Additional file 3.** Review history.

## Data Availability

Inspector is publicly available at https://github.com/ChongLab/Inspector [39] and https://codeocean.com/capsule/9679766/tree [51] under the MIT License. The sequencing data of HG002 were downloaded from GIAB at https://github.com/genome-in-a-bottle/giab_data_indexes, where PacBio 70x (CLR), PacBio CCS 15kb_20kb chemistry2 (HiFi), and Oxford Nanopore ultralong were used for assembly evaluation and error correction, and PacBio CCS 11 kb and 15 kb were used for evaluating assemblies before and after error correction. The benchmark variant callsets used for assembly error validation were downloaded from GIAB [42, 43]. The PacBio CLR dataset and curated genome assembly (Version *assembly_curated* from 26 Sep 2018) of Anna’s hummingbird were downloaded from GenomeArk [52].
