# Supplementary material for: Accurate long-read de novo assembly evaluation with Inspector
Source: Genome Biol. 2021 Nov 14;22:312. doi: 10.1186/s13059-021-02527-4 (PMC8590762; doi:10.1186/s13059-021-02527-4)
Supplement: Supplementary file 1 — Additional file 1: Supplementary Fig. S1-S20. [file 13059_2021_2527_MOESM1_ESM.docx]

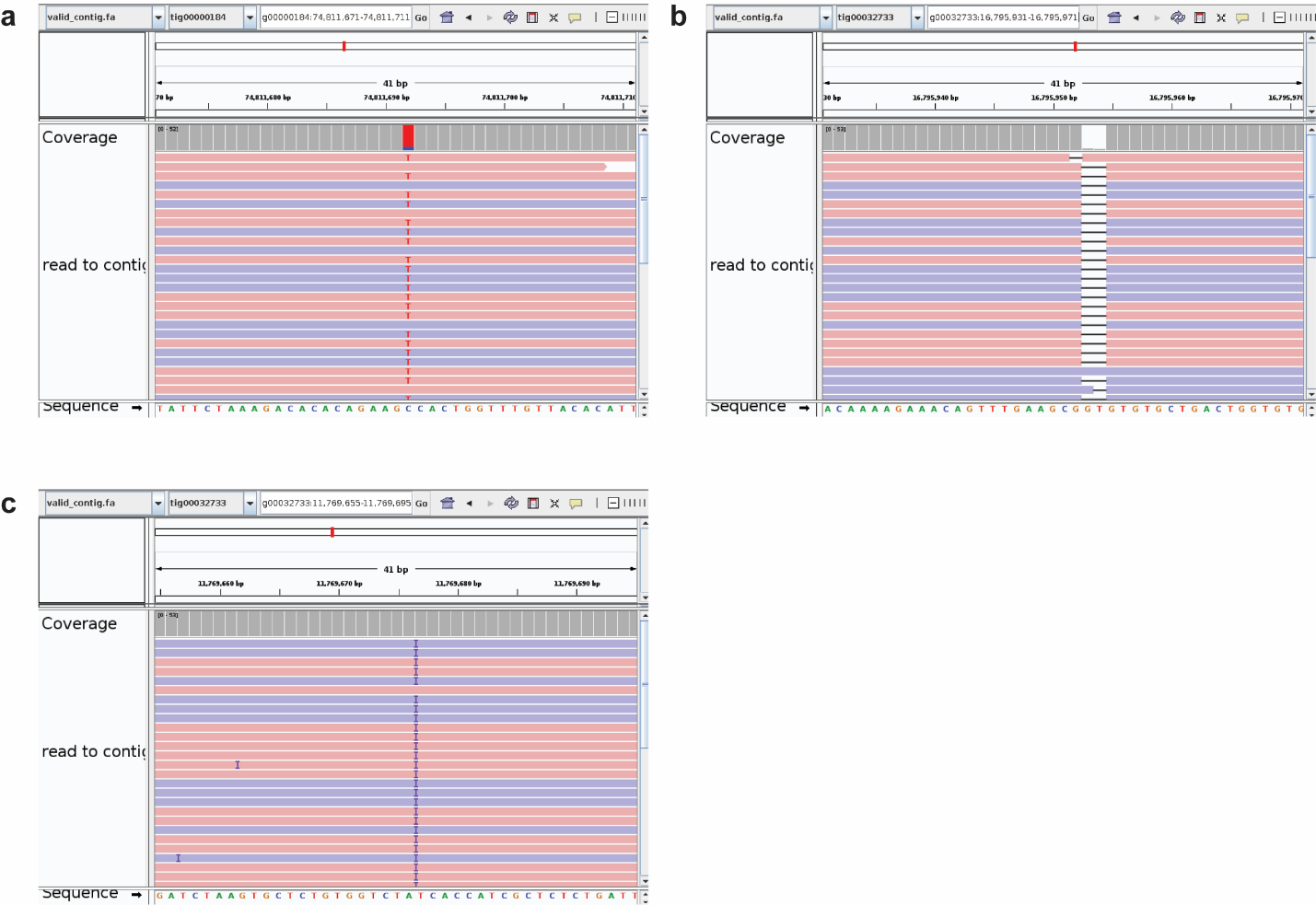


**Figure S1** IGV views of examples of small-scale assembly errors. There are discrepancies between the contig and the majority of reads in base substitution (**a**), small expansion (**b**), and small collapse (**c**).


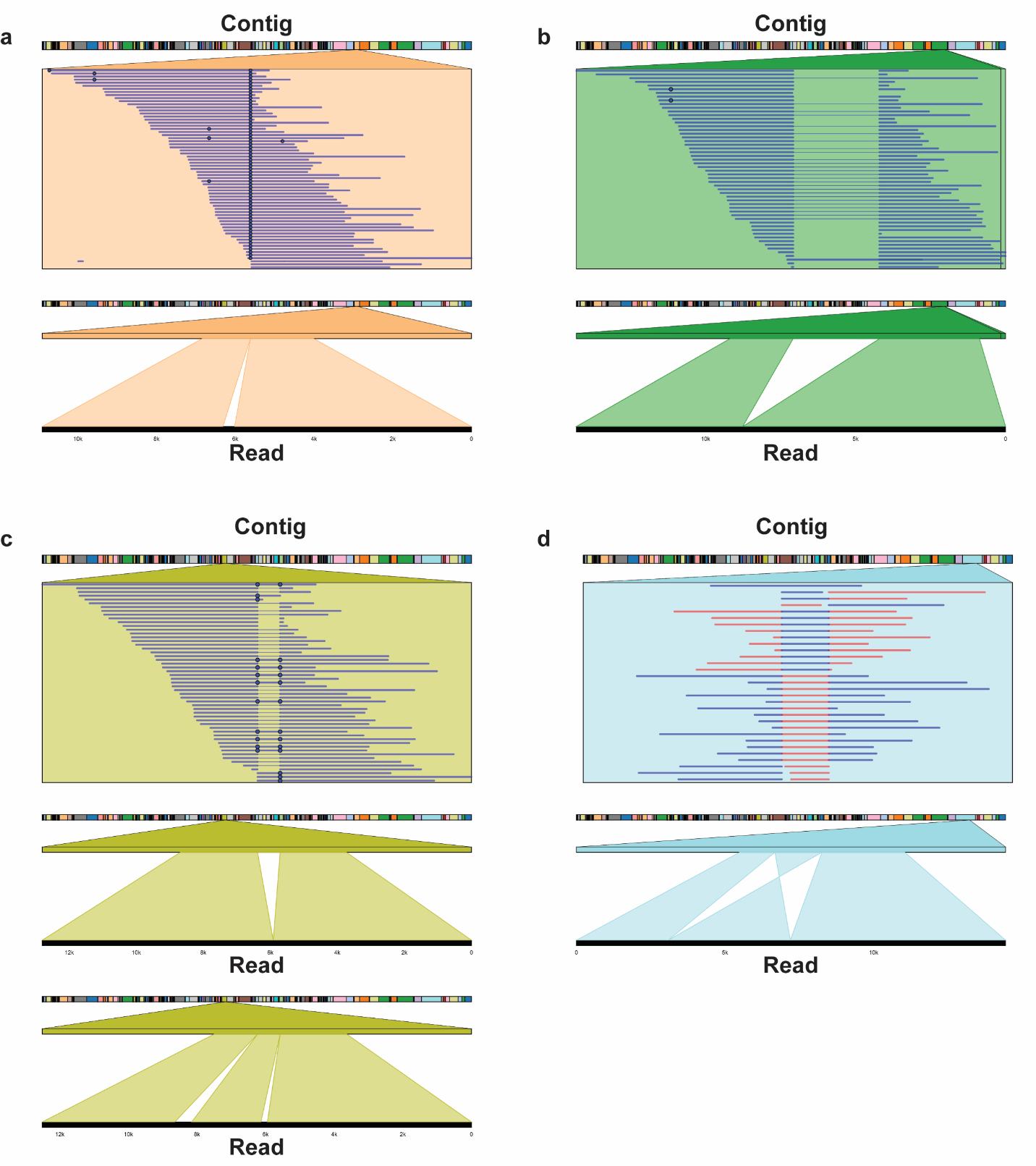


**Figure S2** Examples of structural assembly errors. **a** An insertion-like pattern in read alignment representing a collapse error, as this part of sequence is collapsed in the contig. **b** A deletion-like pattern in read alignment representing an expansion error, as these sequences in contig are expanded and not present in the reads. **c** An insertion-like pattern in half of the reads and a deletion-like pattern in the other half of the reads representing a haplotype switch, as the contig is different from both haplotypes at this heterozygous region. **d** Inverted alignment within reads representing as an inversion error.


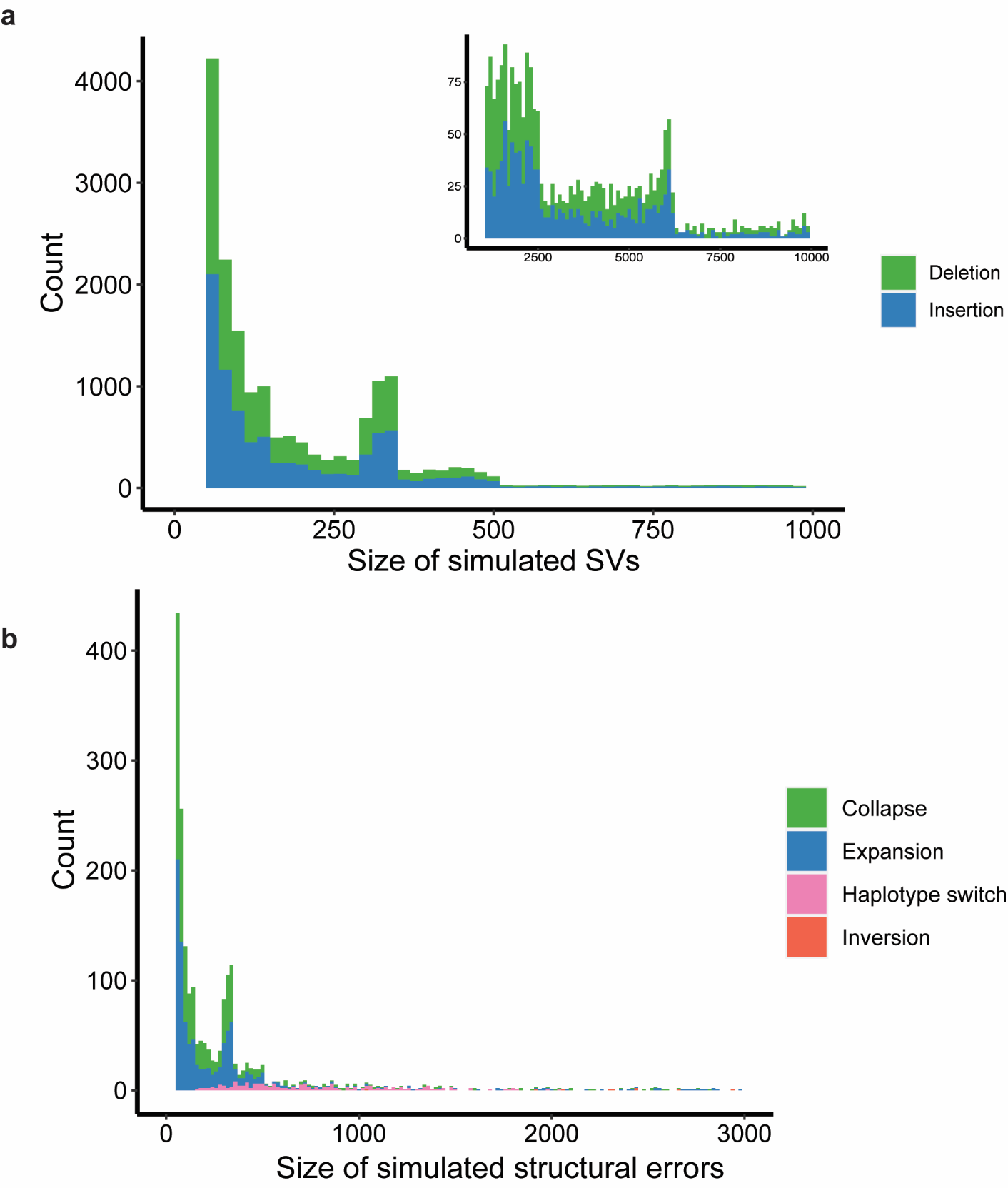


**Figure S3** **a** Size distribution of structural variants in the simulated genome. The peak at ~350bp and ~6kbp were induced to mimic SVs caused by Alu and LINE elements. **b** Size distribution of the simulated structural assembly errors.


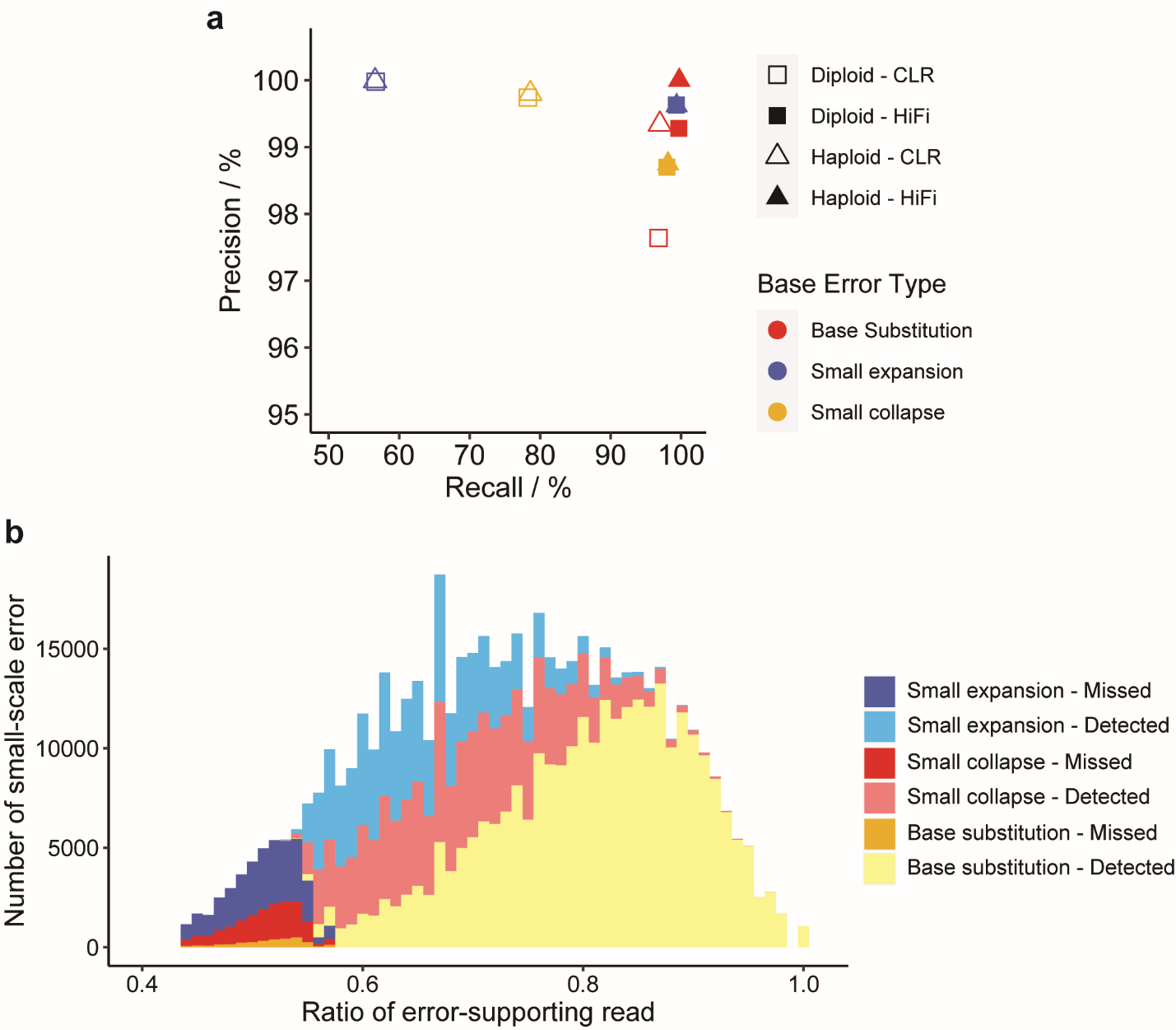


**Figure S4** Small-scale error detection in the simulated dataset. **a** Recall and precision of small-scale error detection. The recall was lower for small expansion and collapse in two CLR datasets. **b** Distribution of ratio of error-supporting read of three subtypes of small-scale errors in Diploid-CLR evaluation. Missed assembly errors showed lower ratio of error-supporting read, owing to the presence of sequencing errors.


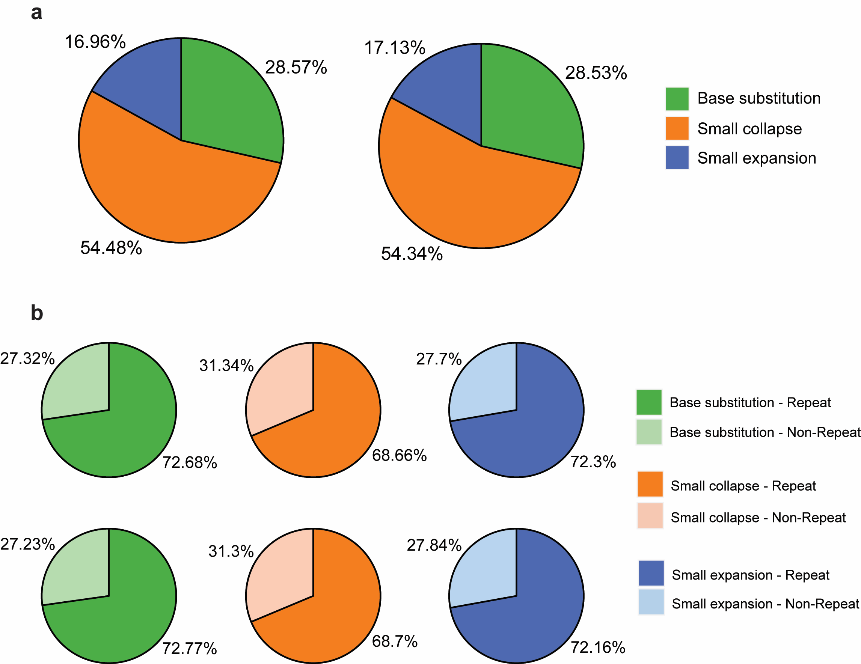


**Figure S5** Small-scale error missed by Merqury but detected by Inspector in the simulated dataset. **a** Three subtypes of small-scale errors detected by Inspector but not by Merqury in haploid (left) and diploid (right) simulation. **b** Composition of Merqury-missed assembly errors located within and outside the repetitive regions for haploid (top) and diploid (bottom) simulation.


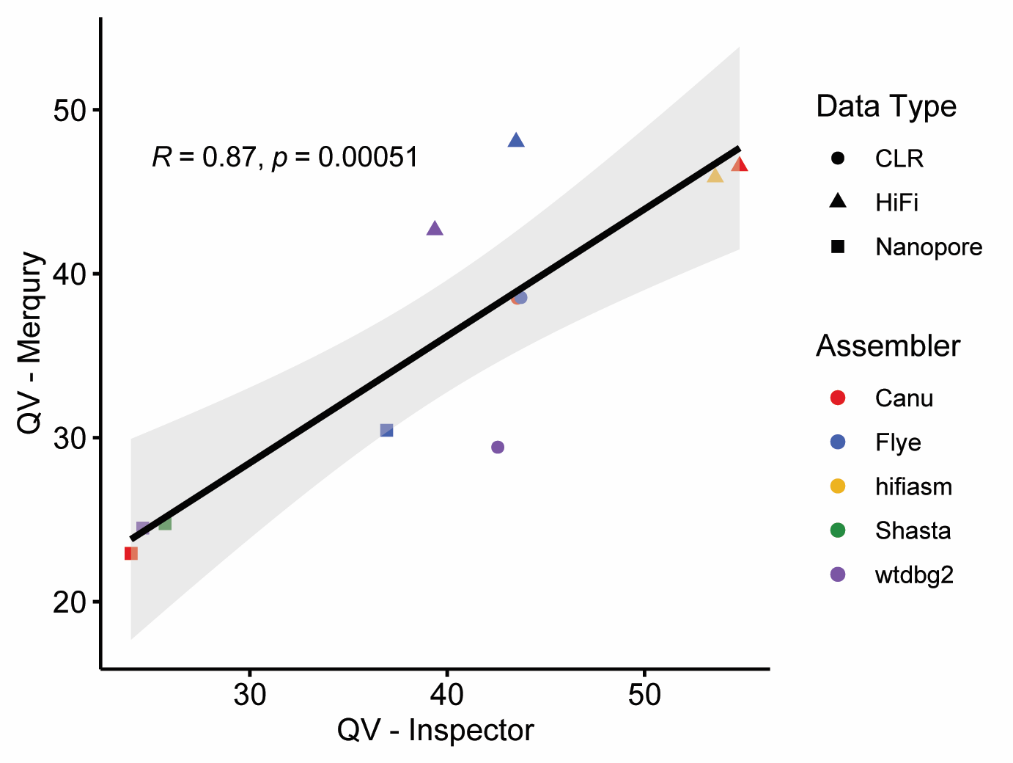


**Figure S6** Correlation between QV scores computed by Inspector and Merqury in all HG002 assemblies.


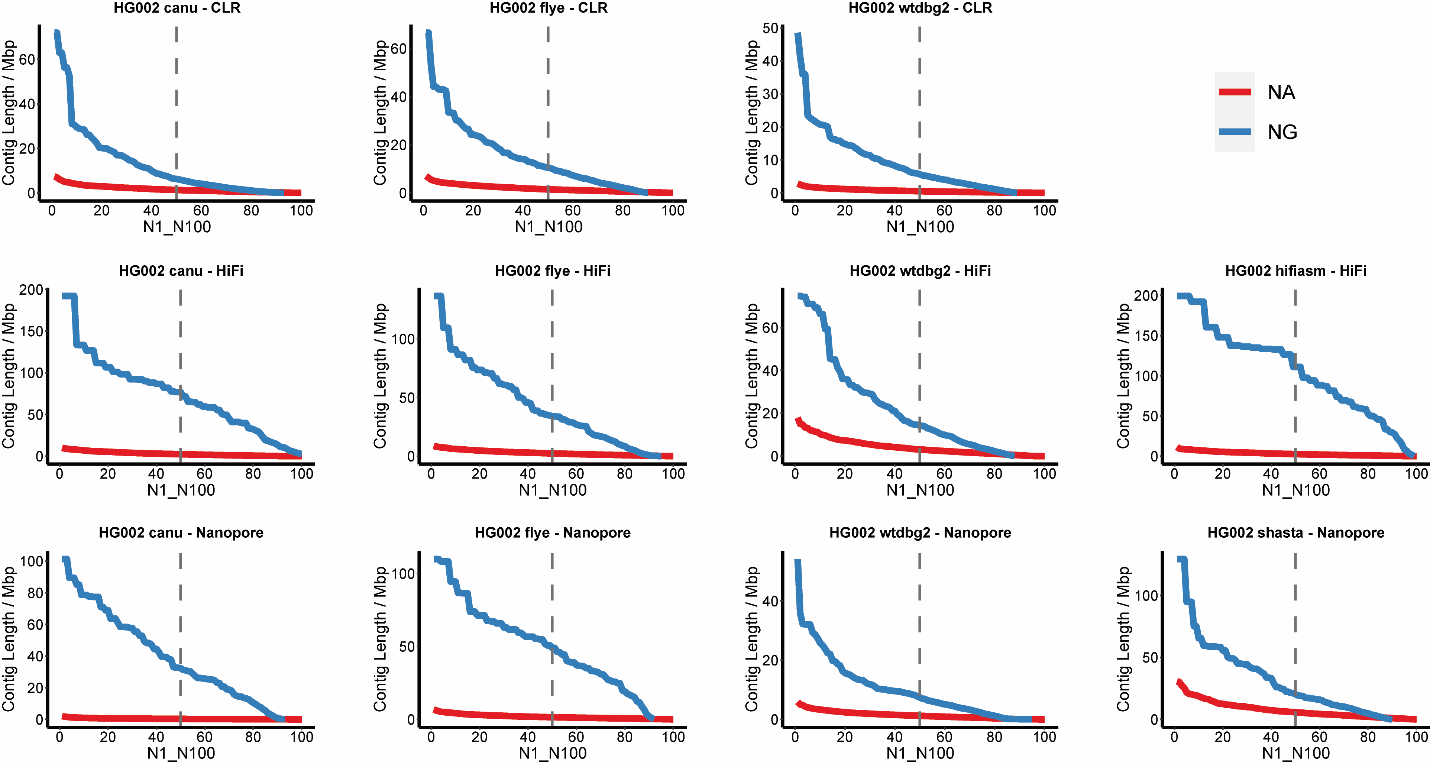


**Figure S7** N1-N100 plot of HG002 assemblies. Dashed lines indicate the NA50 and NG50 at 50% of total assembly length. NAs were calculated on the basis of aligned blocks instead of the contig lengths. NGs were calculated on the basis of known or estimated genome size.


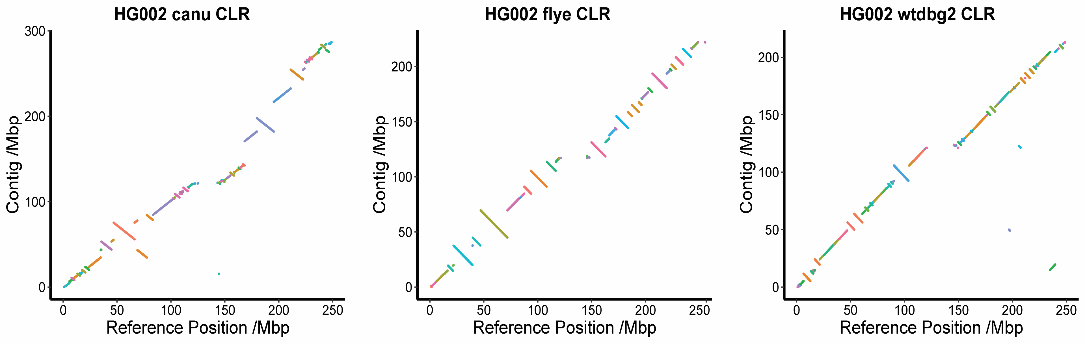

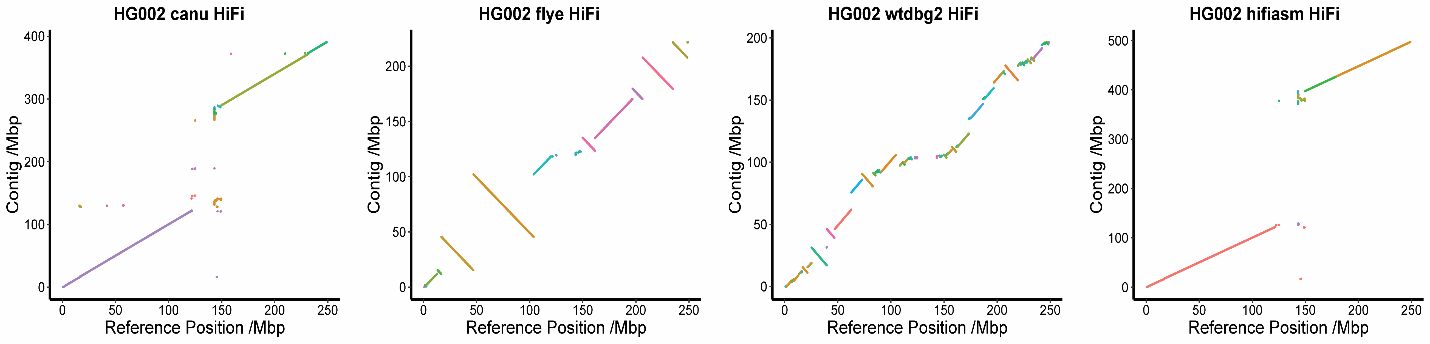

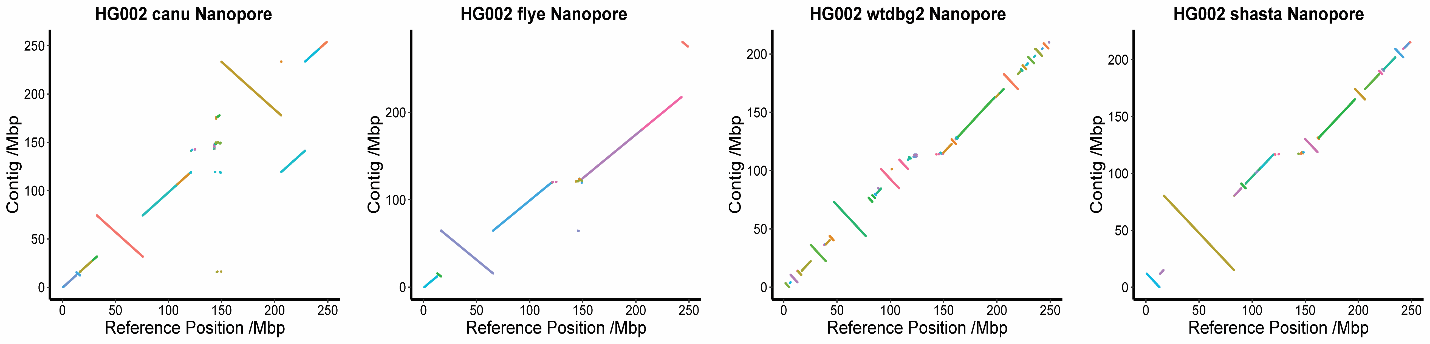


**Figure S8** Dotplot of HG002 assemblies. Each dot represents the base match between contig and the reference genome. Dots from the same contig are marked with the same color.


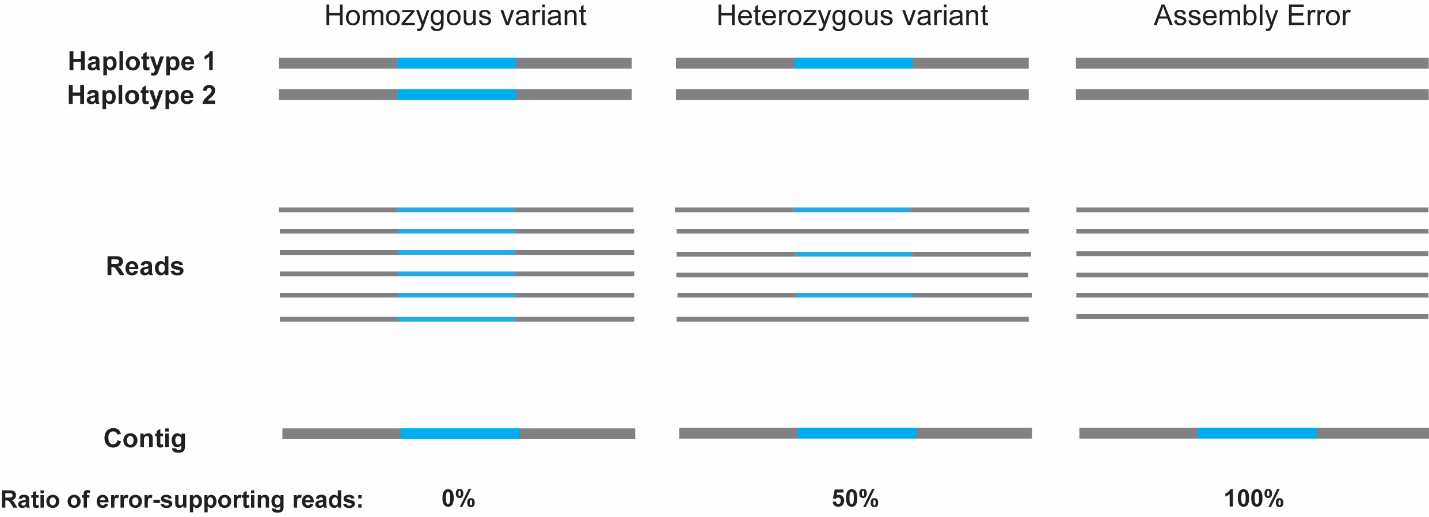


**Figure S9** A theoretical interpretation of the difference between an assembly error and genetic variants in a diploid genome. Sequences differing from the reference genome are marked in blue. For a homozygous variant (left), the contig is consistent with both haplotypes, in which all reads are identical with the contig. In this case, there is no assembly error. For a heterozygous variant (middle), reads from one haplotype are different from the contig, with a ratio of error-supporting reads around 50%. This ratio is close to the frequency of a heterozygote. This is not an assembly error. Only a substantially high ratio (close to 100% theoretically) of reads supporting the error will be considered assembly error (right).


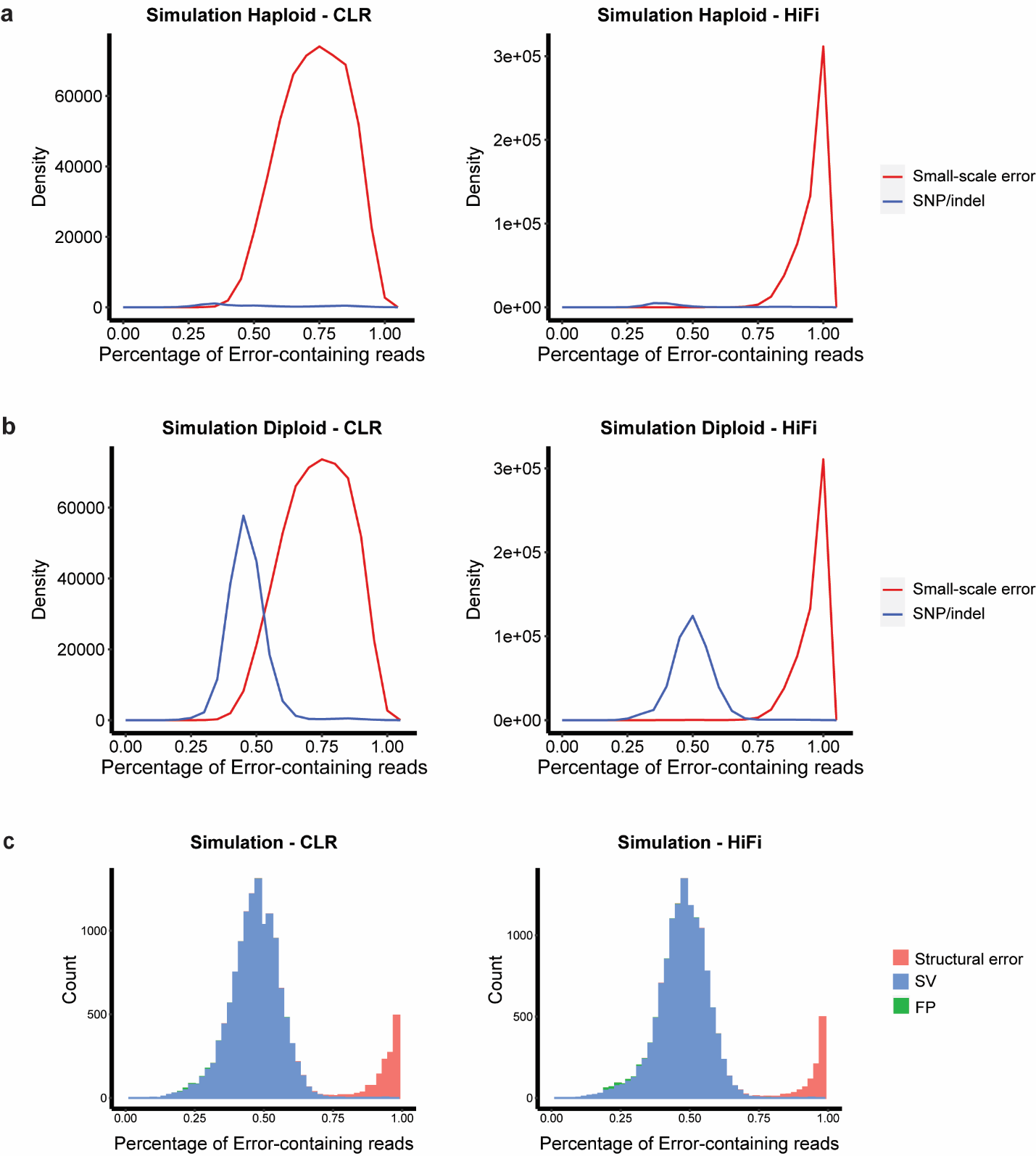


**Figure S10 a,b** Distribution of ratios of error-supporting reads for small-scale assembly errors and SNPs/indels in simulated haploid (**a**) and diploid (**b**) datasets. Small-scale errors are more separate from genetic variants in HiFi datasets than in CLR datasets. **c** Distribution of ratios of error-supporting reads for structural errors. Structural errors show higher ratios than SVs in both CLR and HiFi datasets.


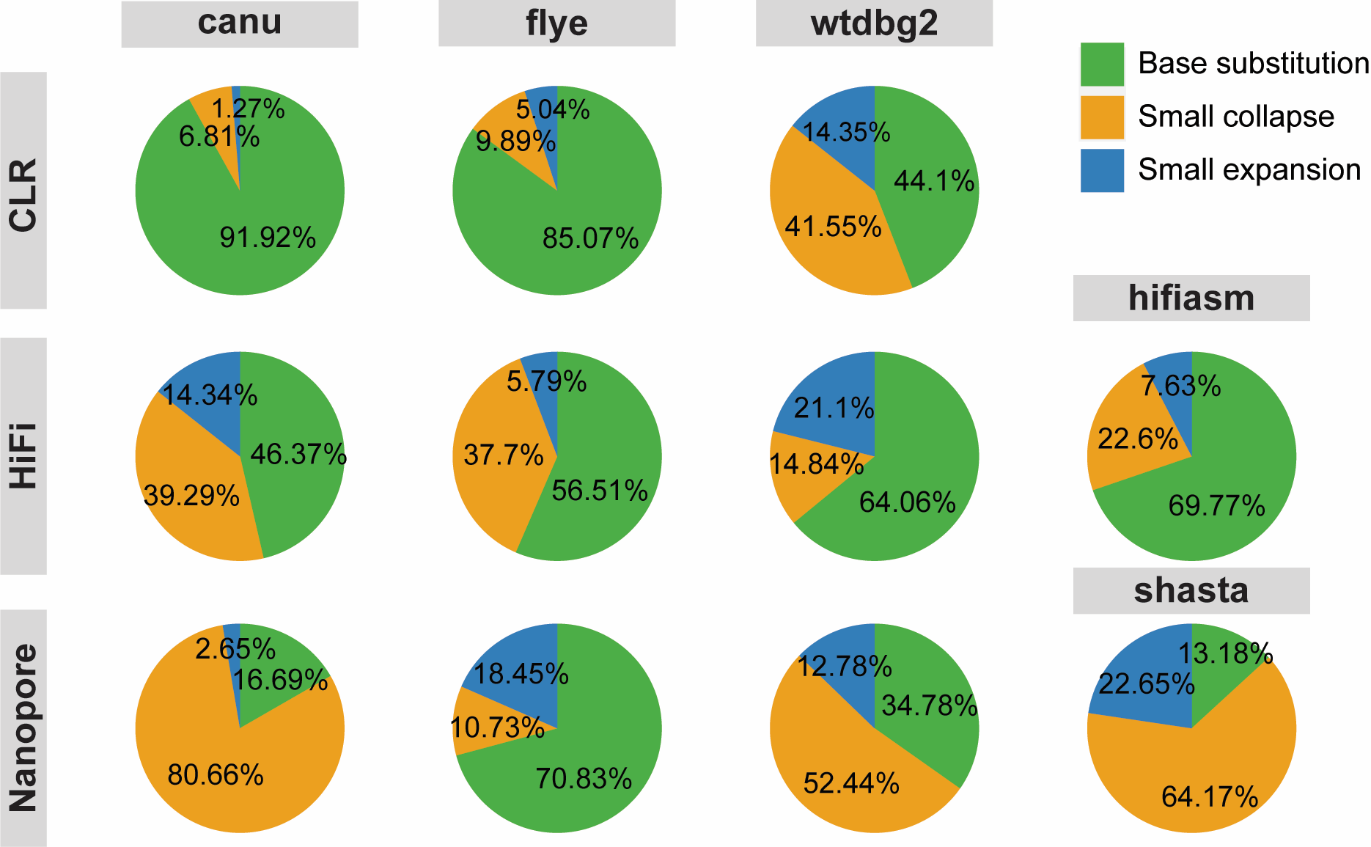


**Figure S11** Pie charts of three types of small-scale errors in HG002 assemblies. The percentage of each error type in total errors is also labeled in each section.


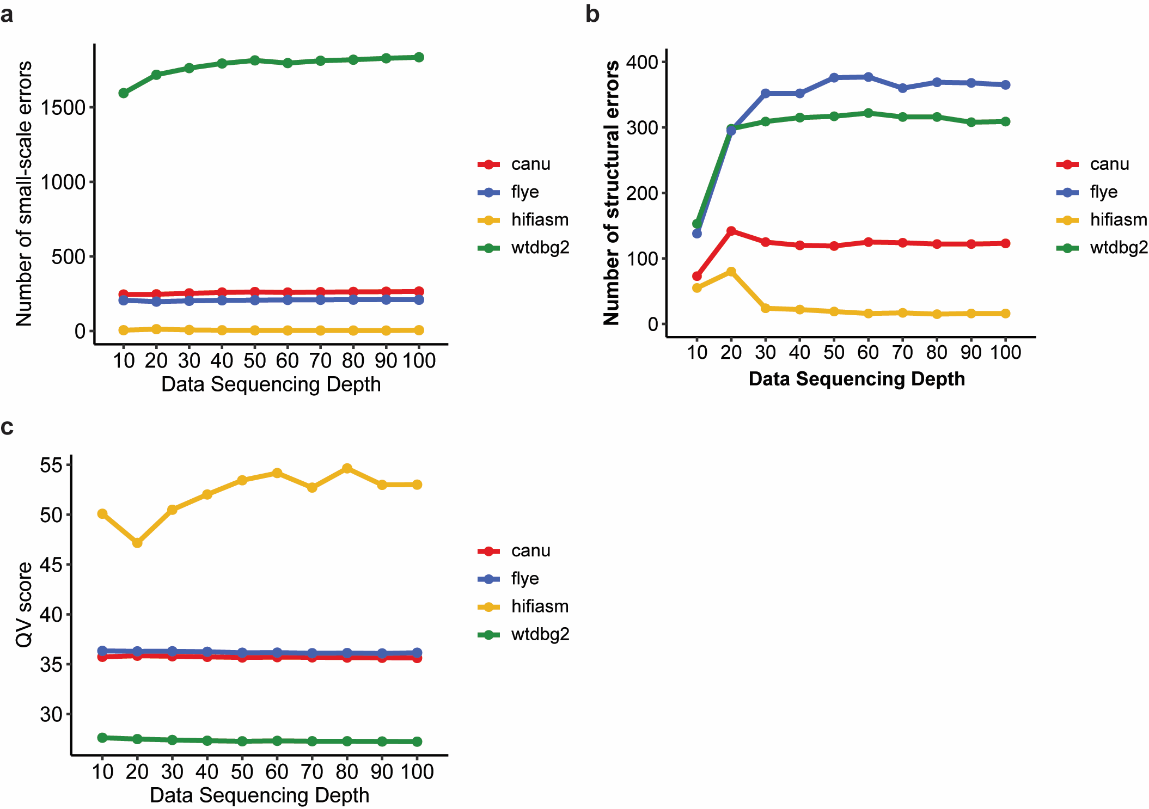


**Figure S12** Inspector evaluation with down-sampled dataset. **a,b** Number of small-scale errors (**a**) and structural errors (**b**) reported from datasets with differing sequencing depth. The numbers of structural errors were fluctuant at 10-20X and stabilized after 30X. **c** QV score at different sequencing depth.


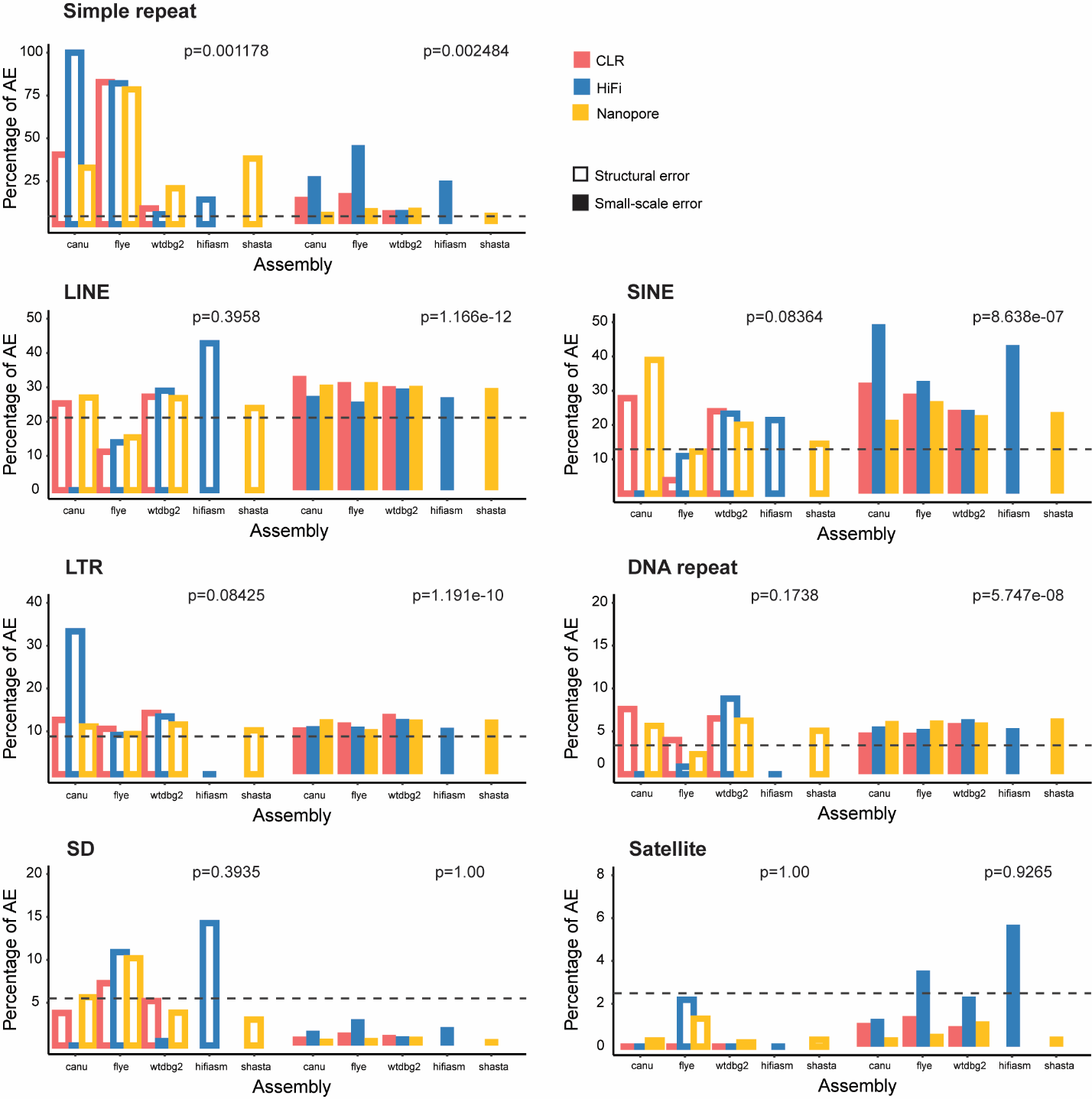


**Figure S13** Proportion of assembly errors located in each type of repeat. P-value (on each panel, left: structural errors, right: small-scale errors) was calculated with one-sample t-test. The dashed line in each plot indicates the percentage of reference genome covered by that repeat type.


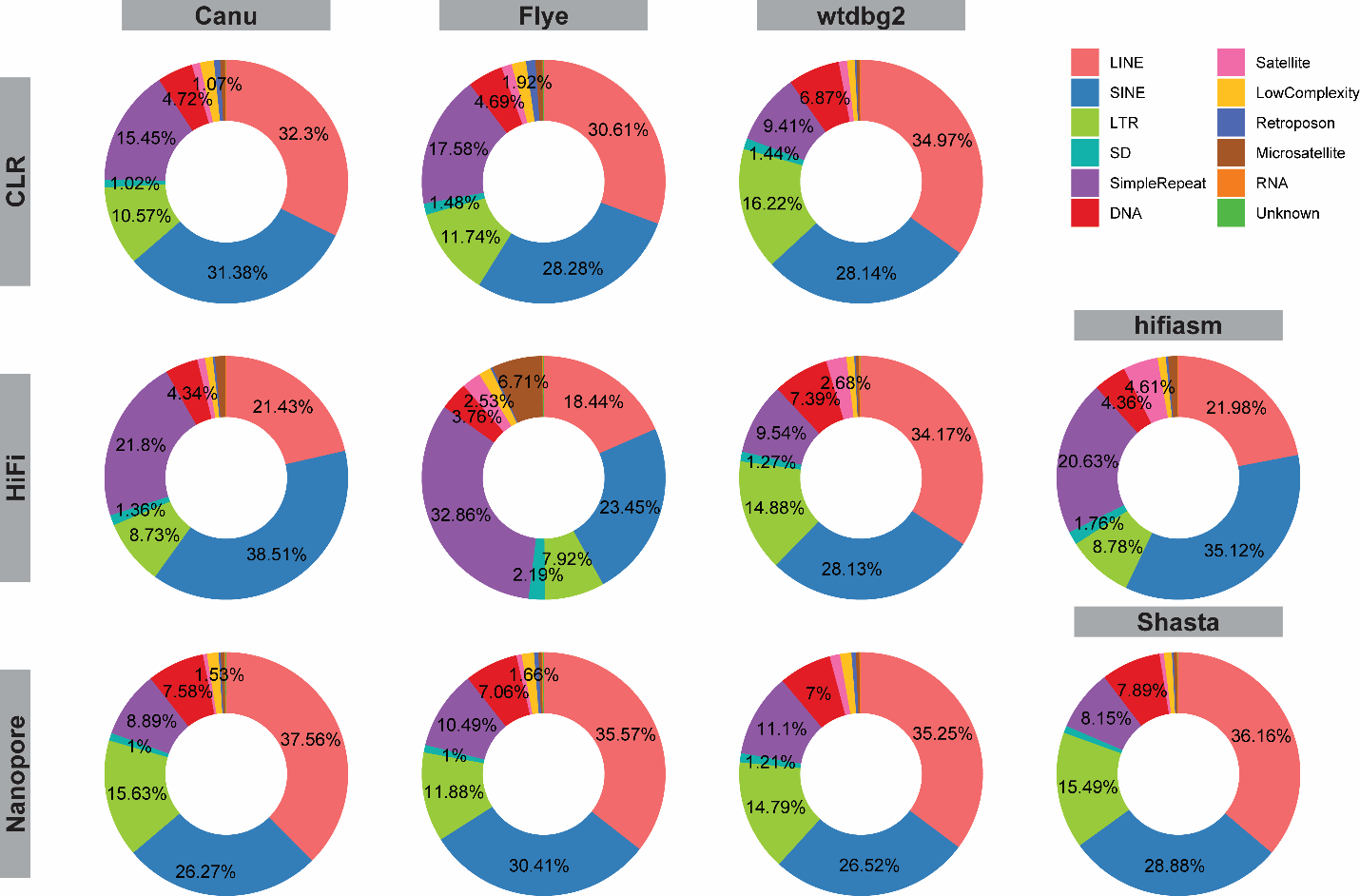


**Figure S14** Repeat annotation for small-scale errors in HG002 assemblies. The proportions of sectors that are larger than 1% are marked in each plot.


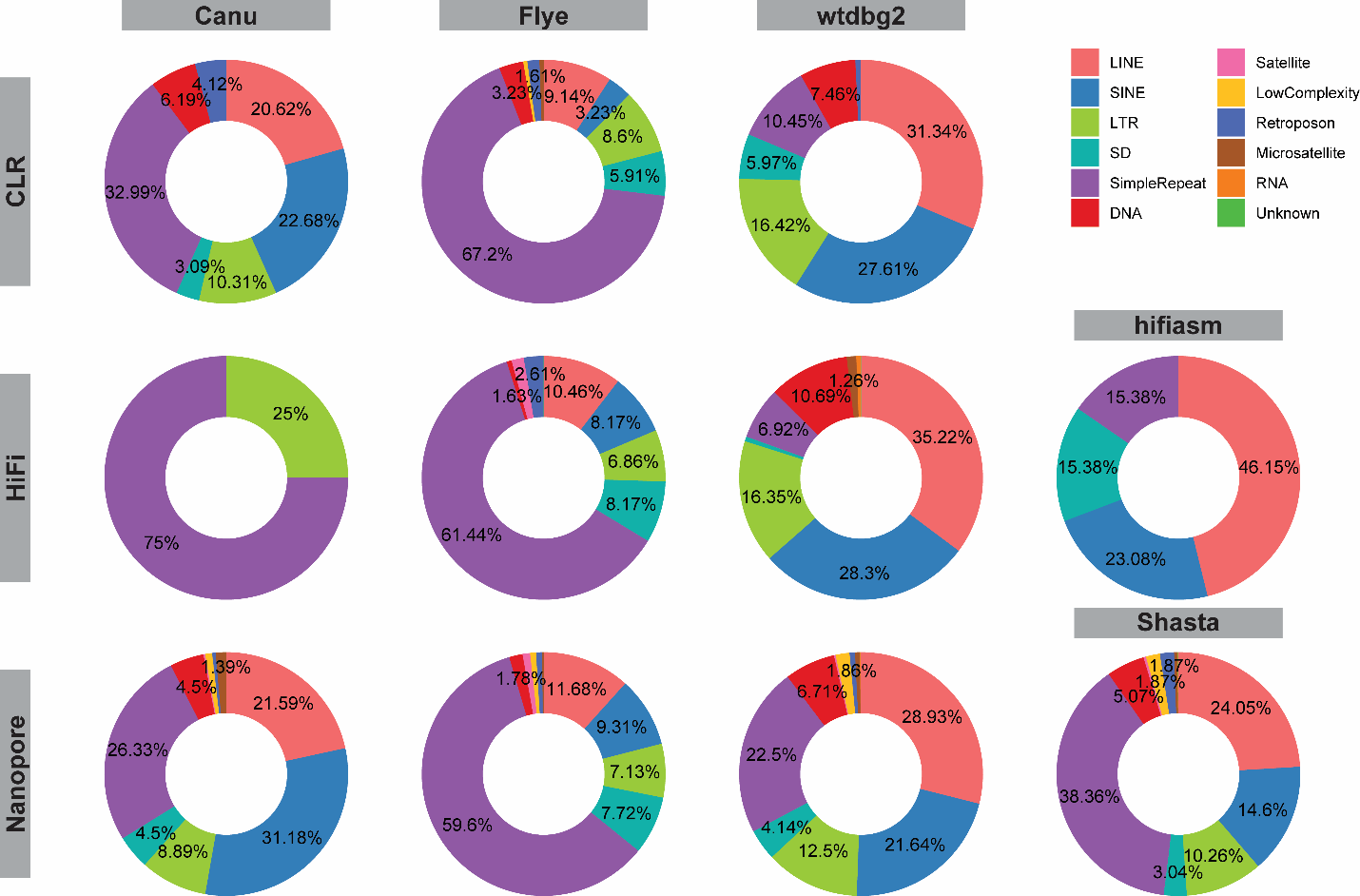


**Figure S15** Repeat annotation for structural errors in HG002 assemblies. The proportions of sectors that are larger than 1% are marked in each plot.


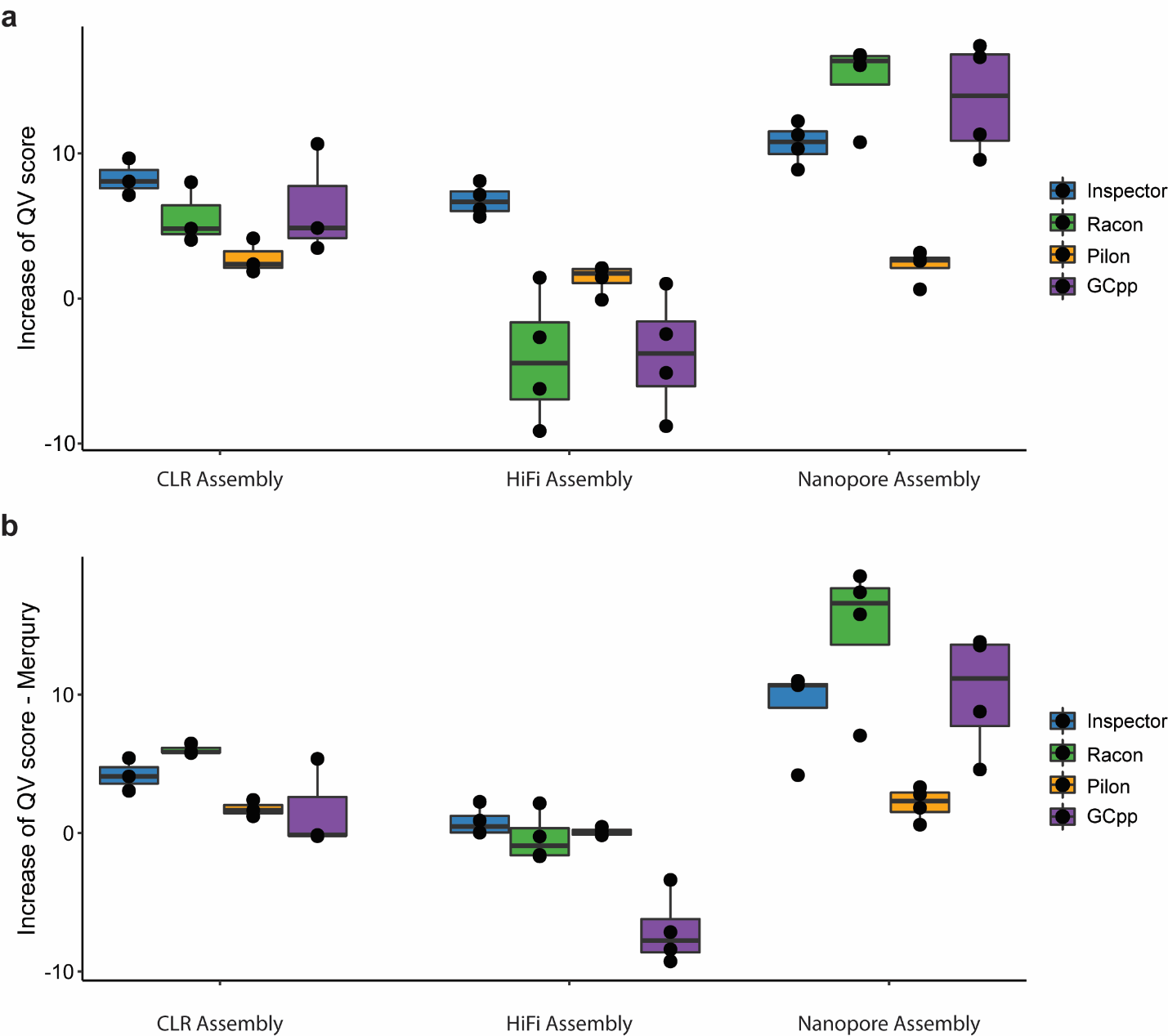


**Figure S16** QV score improvement after polishing with PacBio HiFi reads. **a** QV score of polished assemblies estimated by Inspector. Inspector showed highest improvement in CLR and HiFi assemblies, and Racon showed highest improvement in Nanopore assembly. **b** QV score of polished assemblies estimated by Merqury. Inspector showed best improvement in HiFi assembly, and Racon showed best improvement in HiFi and Nanopore assemblies.


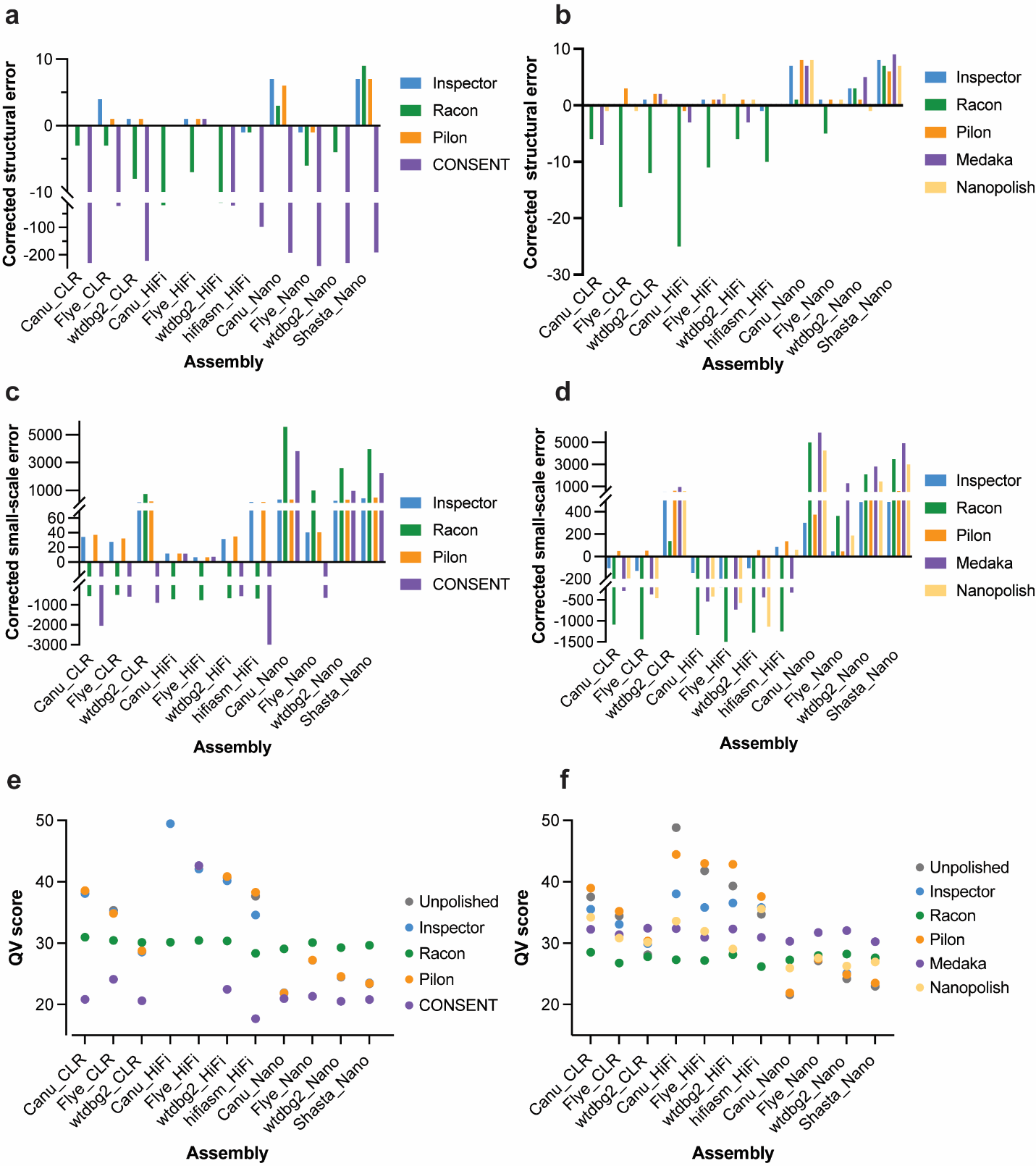


**Figure S17** Assembly error correction with CLR and Nanopore data. **a,b** Number of corrected structural errors after polishing with CLR (**a**) and Nanopore (**b**) data. Inspector fixed most structural errors among tested polishing methods in 9 and 4 out of 11 assemblies in CLR and Nanopore data, respectively. **c,d** Number of corrected small-scale errors after polishing with CLR (**c**) and Nanopore (**d**) data.


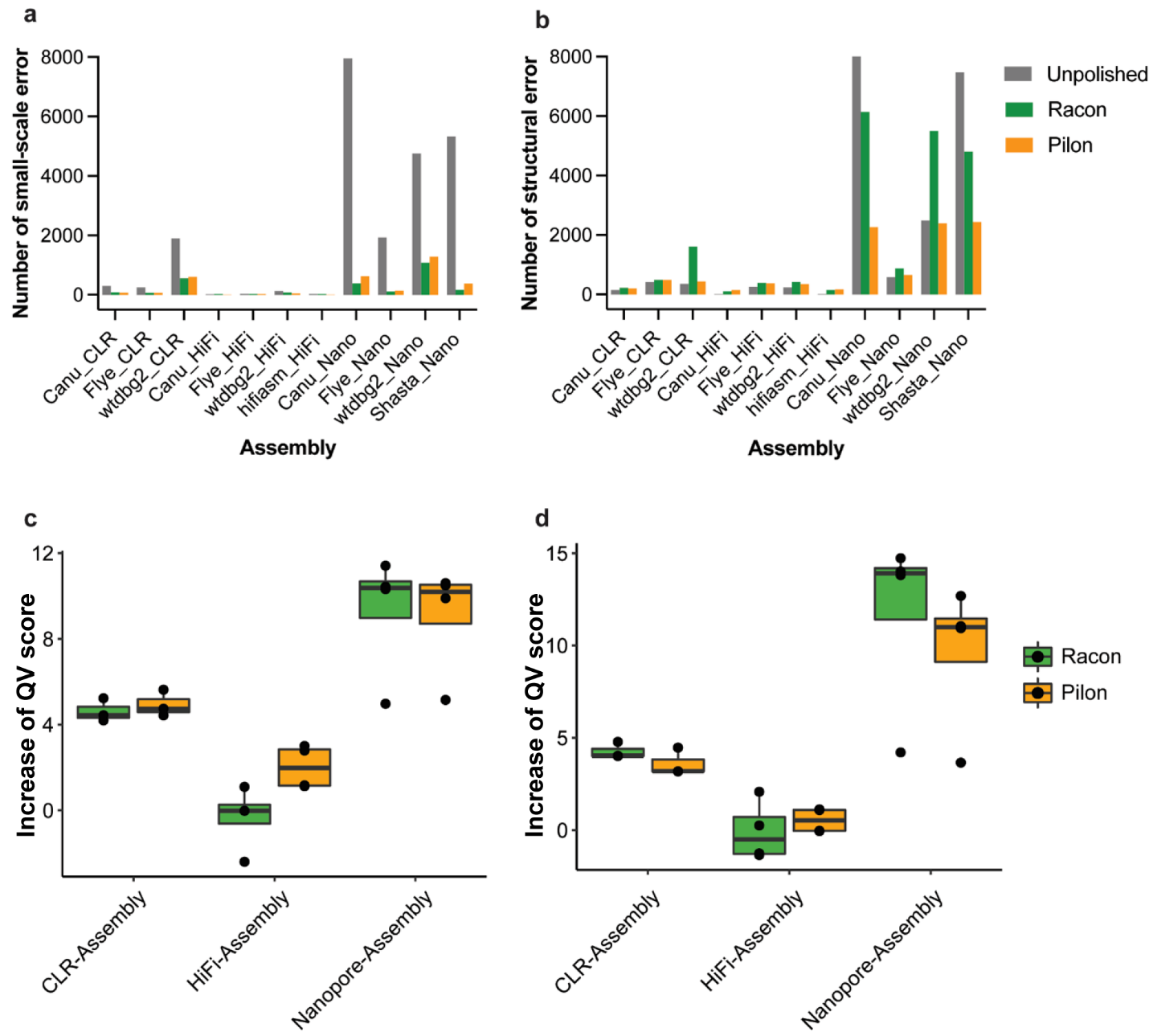


**Figure S18** Polishing HG002 assemblies with Illumina dataset. **a** Number of small-scale errors in the original and polished assemblies. The number of small-scale errors was reduced after short-read polishing with both Racon and Pilon. **b** Number of structural errors before and after polishing with Illumina data. After short-read polishing, the number of structural errors increased in 9 Racon-polished and 8 Pilon-polished assemblies out of 11 total assemblies. **c,d** Improvement of QV scores after short-read polishing process estimated by Inspector (**c**) and Merqury (**d**). The QV scores of CLR and Nanopore assemblies were increased after short-read polishing, while the QV scores of HiFi assemblies showed minor improvement from short-read polishing.


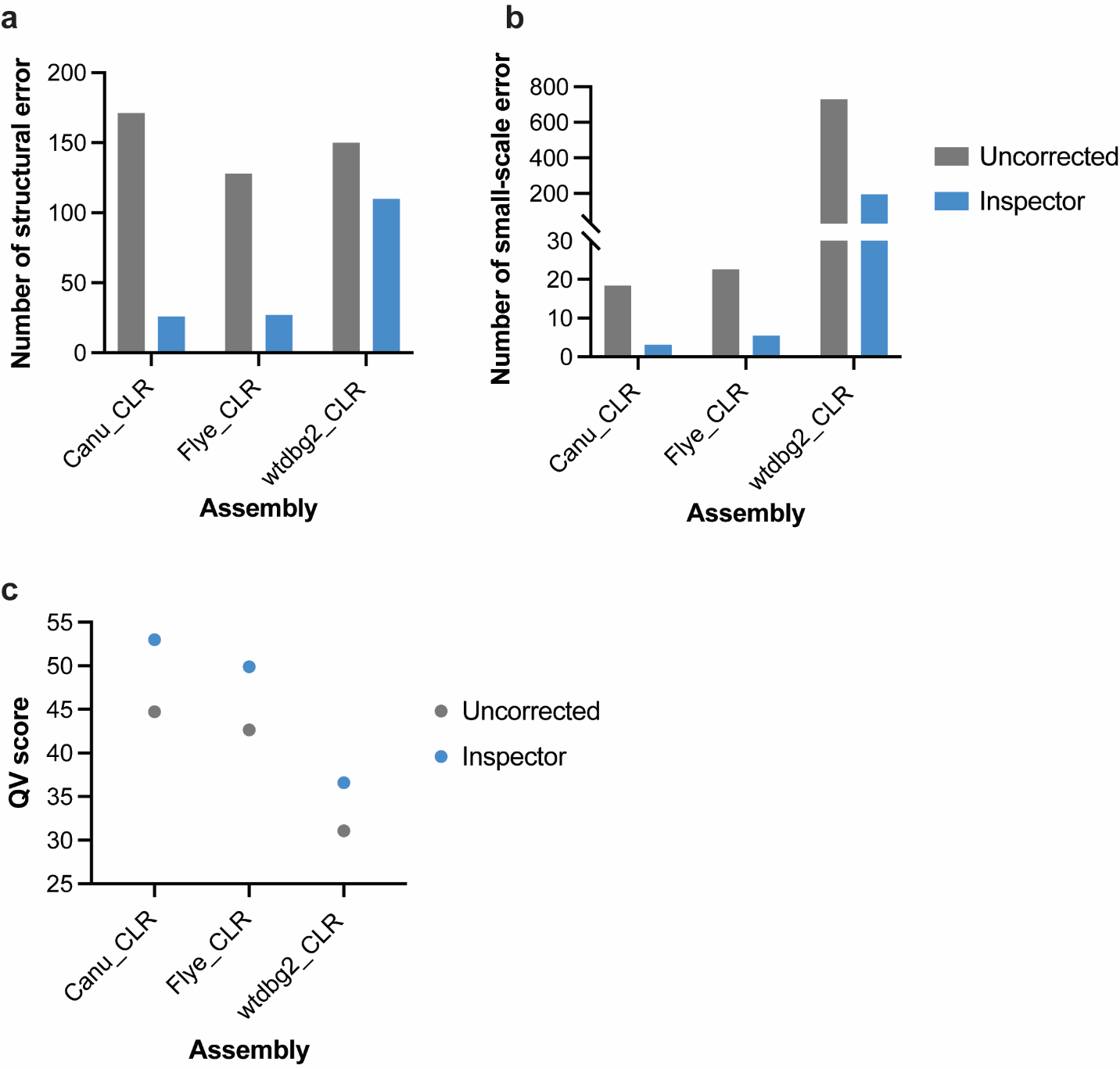


**Figure S19** Inspector error correction in Anna’s Hummingbird genome assemblies. **a,b** Number of structural (**a**) and small-scale (**b**) errors in the uncorrected and Inspector-corrected assemblies. Both structural and small-scale errors dropped after error correction. **c** QV score of uncorrected and Inspector-corrected assemblies. The QV score was increased in all three assemblies.


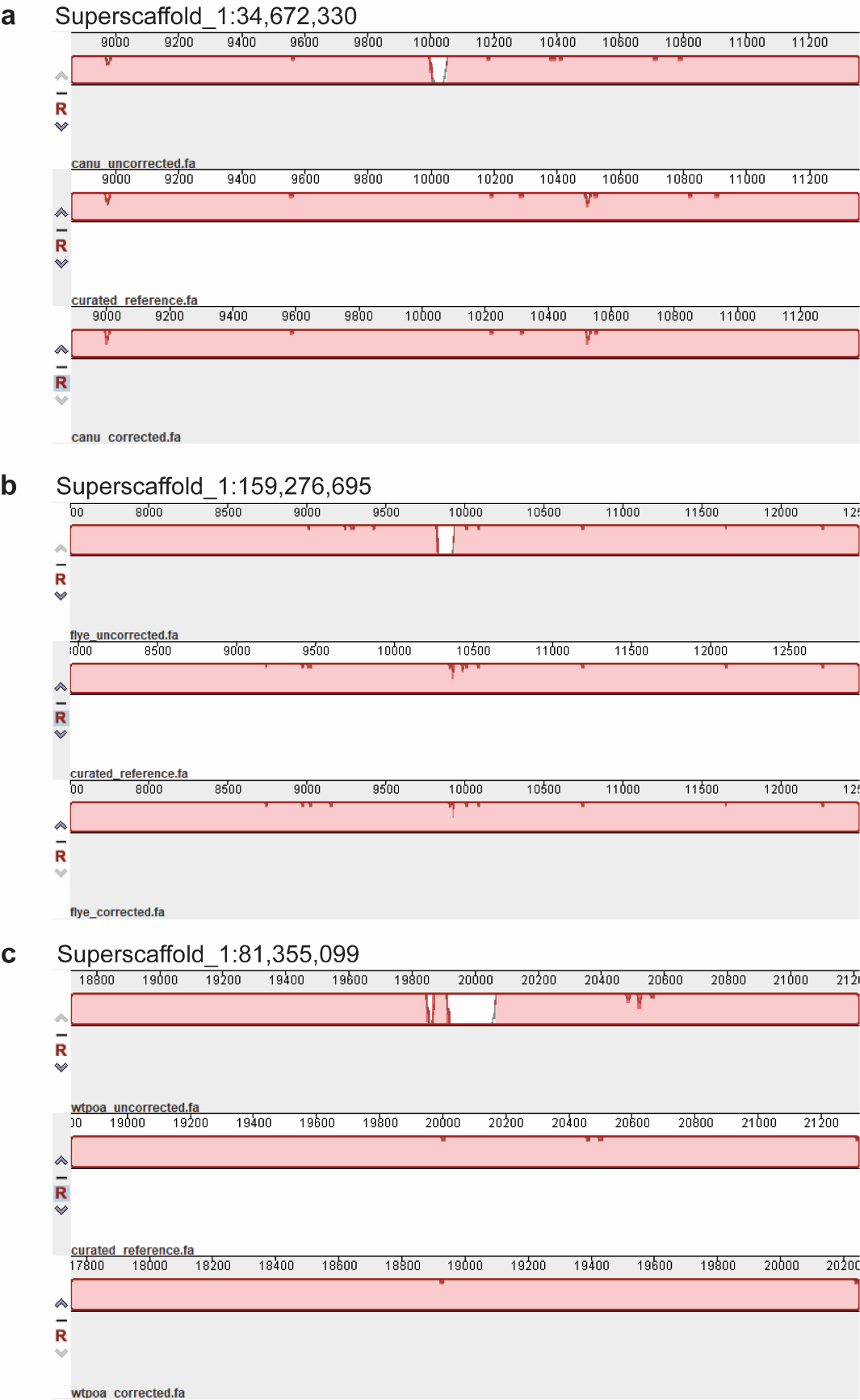


**Figure S20** Example of structural errors corrected by Inspector error-correction module in Canu (**a**), Flye (**b**), and wtdbg2 (**c**) assemblies. The uncorrected contigs (top) showed inconsistency with the curated genome (middle), while the same regions in corrected contigs (bottom) were consistent with curated genome.
