# Supplementary material for: Accurate long-read de novo assembly evaluation with Inspector
Source: Genome Biol. 2021 Nov 14;22:312. doi: 10.1186/s13059-021-02527-4 (PMC8590762; doi:10.1186/s13059-021-02527-4)
Supplement: Supplementary file 2 — Additional file 2: Supplementary Tables S1-S6. [file 13059_2021_2527_MOESM2_ESM.docx]

| **Table S1** Number of assembly errors in the simulated assembly |
| --- |

|  | | | **Haploid** |  | **Diploid** | | |
| --- | --- | --- | --- | --- | --- | --- | --- |
| **Structural** | Expansion |  | 995 |  |  | 900 |  |
|  | Collapse |  | 995 |  |  | 900 |  |
|  | Haplotype switch |  | 0 |  |  | 190 |  |
|  | Inversion |  | 10 |  |  | 10 |  |
| **Small-scale** | Base substitution |  | 289795 |  |  | 289805 |  |
|  | Small expansion |  | 144817 |  |  | 144751 |  |
|  | Small collapse |  | 144715 |  |  | 144771 |  |

| **Table S2** Validation rate of haplotype switch in HG002 assemblies | | | | | |
| --- | --- | --- | --- | --- | --- |
|  |  | **# Haplotype switch** |  | **# Validated** | **Validation rate** |
| **CLR** | Canu | 48 |  | 48 | 100.0 |
|  | Flye | 38 |  | 37 | 97.37 |
|  | wtdbg2 | 44 |  | 44 | 100.0 |
| **HiFi** | Canu | 0 |  | 0 | - |
|  | Flye | 61 |  | 50 | 81.97 |
|  | wtdbg2 | 30 |  | 27 | 90.0 |
|  | hifiasm | 0 |  | 0 | - |
| **Nanopore** | Canu | 370 |  | 357 | 96.49 |
|  | Flye | 119 |  | 108 | 90.76 |
|  | wtdbg2 | 65 |  | 54 | 83.08 |
|  | Shasta | 73 |  | 60 | 82.19 |

*Only haplotype switches located within benchmark regions of HG002 were subjected for validation.

| **Table S3** Small-scale error in HG002 assemblies after error correction | | | | | |  |
| --- | --- | --- | --- | --- | --- | --- |
| **Assembly** | | **Original** | **CLR-corrected** | **HiFi-corrected** | **Nanopore-corrected** | |
| **CLR** | Canu | 299.15 | 17.95 | 85.94 | -21.87 | |
|  | Flye | 245.95 | 19.28 | 86.80 | -32.13 | |
|  | wtdbg2 | 1894.59 | 10.80 | 90.71 | 33.44 | |
| **Total** |  | **2439.69** | **12.53** | **89.73** | **20.05** | |
|  |  |  |  |  |  | |
| **HiFi** | Canu | 15.98 | 75.53 | 87.72 | -840.12 | |
|  | Flye | 36.92 | 40.38 | 76.57 | -394.92 | |
|  | wtdbg2 | 126.50 | 46.49 | 84.37 | -66.91 | |
|  | hifiasm | 29.44 | 82.39 | 89.55 | -457.95 | |
| **Total** |  | **208.84** | **52.69** | **83.98** | **-239.19** | |
|  |  |  |  |  |  | |
| **Nanopore** | Canu | 7952.72 | 7.59 | 95.06 | 10.01 | |
|  | Flye | 1924.54 | 4.34 | 92.83 | 3.99 | |
|  | wtdbg2 | 4754.68 | 11.76 | 91.00 | 20.74 | |
|  | Shasta | 5329.15 | 8.74 | 94.55 | 10.70 | |
| **Total** |  | **19961.09** | **8.58** | **93.74** | **12.17** | |

*The number of small-scale errors in the original assembly are listed in the ‘Original’ column and the unit is Mbp^-1^. The error correction rate after correction with CLR, HiFi, and Nanopore data are listed in the other columns. A negative error correction rate indicates more small-scale errors after error correction. The total small-scale errors and error correction rates for each type of assembly are shown in bold.

| **Table S4** Structural error in HG002 assemblies after error correction | | | | | |  |
| --- | --- | --- | --- | --- | --- | --- |
| **Assembly** | | **Original** | **CLR-corrected** | **HiFi-corrected** | **Nanopore-corrected** | |
| **CLR** | Canu | 146 | 64.38 | 58.22 | 29.45 | |
|  | Flye | 407 | 43.49 | 49.63 | 18.43 | |
|  | wtdbg2 | 351 | 45.30 | 27.35 | 24.50 | |
| **Total** |  | **904** | **47.57** | **42.37** | **22.57** | |
|  |  |  |  |  |  | |
| **HiFi** | Canu | 4 | -50.00 | -150.00 | -100.00 | |
|  | Flye | 257 | 18.68 | 68.87 | 15.18 | |
|  | wtdbg2 | 236 | 15.25 | 58.47 | 13.98 | |
|  | hifiasm | 16 | 37.50 | 62.50 | -6.25 | |
| **Total** |  | **509** | **17.15** | **62.18** | **13.06** | |
|  |  |  |  |  |  | |
| **Nanopore** | Canu | 9622 | 62.72 | 76.12 | 60.06 | |
|  | Flye | 582 | 20.45 | 12.54 | 40.03 | |
|  | wtdbg2 | 2484 | 20.25 | 29.39 | 30.23 | |
|  | Shasta | 7469 | 95.43 | 55.66 | 48.87 | |
| **Total** |  | **10535** | **68.39** | **60.94** | **51.66** | |

*The number of structural errors in the original assembly are listed in the ‘Original’ column. The error correction rate after correction with CLR, HiFi, and Nanopore data are listed in the other columns. A negative error correction rate indicates more structural errors after error correction. The total structural errors and error correction rates for each type of assembly are shown in bold.

| **Table S5** Runtime and memory usage of assembly evaluation | | | |
| --- | --- | --- | --- |
|  | **Inspector** | **Merqury** | **QUAST-LG** |
| CPU x node | 12x1 | 12x1 | 12x1 |
| Wall-clock time | 13:33:28 | 07:11:29 | 00:39:38 |
| CPU time | 5-14:10:35 | 3-07:26:48 | 01:09:14 |
| Peak Memory (GB) | 36.34 | 289.76 | 36.19 |

The runtime and memory usage were for evaluating HG002 Canu assembly from CLR reads. Inspector evaluated the assembly with 50x HiFi dataset. Merqury evaluated the assembly with 50x Illumina dataset. QUAST-LG used GRCh38 as the reference genome. The total runtime and memory usage of Merqury includes both meryl database construction and Merqury evaluation.

| **Table S6** Runtime and memory usage of genome polishing | | | | | | | |  |  |
| --- | --- | --- | --- | --- | --- | --- | --- | --- | --- |
|  | | **Inspector** | **Racon** | **Pilon** | **GCpp** | **Medaka** | **Nanopolish*** | **CONSENT*** | |
| CPU x node | | 12x1 | 12x1 | 12x1 | 12x10 | 12x1 | 12x1 | 12x1 | |
| Wall time | | 00:25:58 | 17:40:12 | 03:38:47 | 1-13:03:17 | 1-04:30:55 | 1-10:40:45 | 00:05:52 | |
| CPU time | | 01:30:25 | 7-07:14:25 | 04:08:50 | 17-23:02:25 | 1-23:07:53 | 17-06:33:14 | 00:43:56 | |
| Peak memory (GB) | | 17.40 | 30.35 | 273.22 | 70.42 | 325.31 | 38.13 | 1.86 | |

All polishing methods were tested on one node (12 CPUs) with number of threads set to 12, except for GCpp which was tested on 10 nodes with 12 CPUs per node.

*Nanopolish and CONSENT were tested on only one contig (10Mbp in length).
