# Supplementary material for: Accurate long-read de novo assembly evaluation with Inspector
Source: Genome Biol. 2021 Nov 14;22:312. doi: 10.1186/s13059-021-02527-4 (PMC8590762; doi:10.1186/s13059-021-02527-4)
Supplement: Supplementary file 3 — Additional file 3. Review history. [file 13059_2021_2527_MOESM3_ESM.docx]

Review History

**First round of review**

**Reviewer 1**

**Were you able to assess all statistics in the manuscript, including the appropriateness of statistical tests used?**

Yes

**Were you able to directly test the methods?**

Yes

**Comments to author:**

Authors describe the method Inspector, a tool with two main functions: (i) assembly evaluation and (ii) correction. Both of these functions are described in depth in the paper methodology. However comparison of the performance evaluation is done only for the "assembly evaluation" module of Inspector. IMO, the title of the paper is a bit misleading since it also does polishing.

-One of the major challenges of the assembler is resolving repeat regions. Although authors acknowledge the effect of repeats on the assembly errors. But, there is no mention of how these errors are handled either by assembly evaluation or correction modules.

-There is no comparison against state-of-the-art assembly correction/polishing methods and there is no mention of any such methods anywhere in the paper. Since the correction/polishing of the draft assemblies is an integral part of the paper, it is important to have a comprehensive comparison against the available methods. Nanopolish, racon, medaka, Quiver and CONSENT are some of the tools that can do assembly polishing with long reads. Comparison against existing tools is needed and will greatly improve the manuscript. I also suggest that authors cite the state-of-the-art (of assembly correction) in the introduction of the paper.

Correction method links:

CONSENT: https://doi.org/10.1038/s41598-020-80757-5

Quiver: https://doi.org/10.1038/nmeth.2474

racon: http://www.genome.org/cgi/doi/10.1101/gr.214270.116

Medaka: https://nanoporetech.github.io/medaka/

Nanopolish: https://doi.org/10.1038/nmeth.3444

-P8.21.Table2: Caption lists the columns in the table. But, it is difficult to follow which description belongs to which column.

-P12.44: "Patterns" on different datasets should be elaborated.

-P18.19: The rationale behind using binomial distribution to correct small-scale errors should be explained.

- regarding the previous point, inspector should be able to evaluate the assembly of species without reference genome. Now, there is an assumption that genome will be diploid which might not be correct.

-Inspector runs in the CodeOcean capsule as described in the paper. A short summary of the experiment, describing the input dataset and outputs, in the CodeOcean may help the evaluation. Although we understand that CodeOcean is in testing as of yet.

**Reviewer 2**

**Were you able to assess all statistics in the manuscript, including the appropriateness of statistical tests used?**

Yes: I did not redo the experiments to check all numbers. However, the only two statistical tests used (binomial and FDR) make sense and are appropriate.

**Were you able to directly test the methods?**

No

**Comments to author:**

The method addresses the lack of tools to evaluate the accuracy in genome assembly using long reads (LR) and without reference. The authors propose a simple yet effective way of identifying potential errors in de novo genome assemblies.

I have a few comments for the authors to consider:

- One paragraph is duplicated (lines 181 - 189)

- Inspector is designed to work on LR-only datasets, with most success on HIFI data. Have the users tried to assess how much gain, if any, would there be if an additional Illumina short-read dataset is available? Like the authors have said, it is becoming the norm for people to use HIFI-only data for assembly. However, it would be good to see hard evidence once and for all to make an informed decision that the magnitude of gain is not worth the investment to produce short-read datasets on top of LR, or not.

- LR de novo genome assembly is gaining a lot of tractions in discovering and studying plants and animals. Have the authors assessed the performance of Inspector on polypoidy genomes and other "difficult" genomes? If Inspector is designed to work only on haploids and diploids (which would limit its potential, in my opinion), it should have been made clearer.

- Would the authors consider comparing with other common polishing tools (e.g. Pilon, Arrow, Racon,....) and assess performance of individual tools and/or combos of tools in correcting assembly errors?

- Table 1: have you analyzed the characteristics of Inspector's false negatives? Why and how are they missed? e.g. Authors commenting that is "possibly because of the predominance of indel", but obviously Inspector can deal with some indels but not the others. Perhaps they can look at whether the FNs are all sporadic or there are some characteristics that would allow them to be caught, or are there any further explanations as to why they are missed (e.g. missing those are particularly long in length? particular difficulty around centromere?)

- Table 1: similar to above, what are the assembly errors that are missed by Merqury but not Insepctor?

- Line 210: I might have missed it but I can't find detailed descriptions of what filters are applied on structural errors. For small-scale errors, the binomial test is clear. Is it possible to apply an appropriate statistical test for the structural errors as well, instead of an arbitrary filter which can be hard to judge what threshold one should use?

- Any comment on actual run time and hardware requirement?

**Reviewer 3**

**Were you able to assess all statistics in the manuscript, including the appropriateness of statistical tests used?**

Yes: The authors made a clever use of teh binomial distribution to distinguish errors from putative genomic variants (SNVs, SVs & Indels). However, I still have my concerns that its application to estimate Consesus Quality Values is robust enough. I think the authors should clarify some points and test the effect of the mean quality of the raw reads to estimate QV by comparison against both unpolished and polished assemblies (using illumina data).

**Were you able to directly test the methods?**

No

**Comments to author:**

Dear Chen et al,

This is a good work and the manuscript is really well written. I think Inspector could be a very valuable tool for miss-assembly detection in de novo assembled genomes. The program is easy to install following the github link provided. In principle, could be used to distinguish between true variants and error. This would be particularly interesting to detect regions that are heterozygous for (SVs) in de novo assembled genomes. Thus, opening the door to study their biological implications. Your team made a clever use of the binomial distribution to both detect errors (at small and large scale) and apply these methods to identify errors in different assemblies obtained with long-reads of decreasing error rate (nanopore, pacbio CLR and Hifi reads). In addition, the results on the human (HG002) datasets are promising. However, I still have some concerns about the reliability of the methods used, the interpretation of the results and the application of the tool to improve newly assembled genomes. For these reasons I would reject the present version of the manuscript until you address my main concerns. I will be happy to review it again after you revise the current version of the manuscript. I hope the comments below help to strengthen Inspector and improve its applicability.

Sincerely,

Anonymous reviewer

Major Concerns

1. The estimation of Quality Value scores shown here infers the error rate from noisy reads to contigs from long-read only assemblies. In my opinion the estimate error rate is a mixture of both the errors coming from the reads and the assemblies. I think the right way to do this would be using high-accuracy reads against the assemblies (e.g. Merqury paper). Could you explain why you decided to do so?

2. The QV scores are higher at low coverage (Figure S11 c), what is the reason for this? It looks like with lower coverage you are just having less power to detect enough errors in the assemblies. Your equation in fact does not weight the estimates by depth. Therefore, at 10x you detected less errors and the QV looks higher than expected. In my opinion a way to overcome this would be to divide the errors by the total number of bases covered above certain threshold depth (≥ 5x). Uncovered bases and Ns should not be considered in the denominator (p.9). Do you agree?

3. Figure S11 b shows a higher number of Structural errors produced with Flye 2.8.2 using HiFi reads. Do you have any explanation for this?

4. Shasta normally to produce shorter assemblies than all the other assemblers, however the amount of collapse errors using nanopore is a lot lower than for wtbg Are the collapse errors detected in Shasta assemblies spanning larger regions? What is the cumulative length of the collapsed errors compared to other assemblers?

5. Again, about the QV estimates. In the methods you wrote that you used Merqury (Rhie et al. 2020) and you built meryl databases from a fasta. Was this a "reads" fasta or a reference genome (GRCh58)?, what have you used?

6. If you used reads, the method is meant to use high-accuracy reads at enough coverage to estimate the sequence accuracy in the assembly. The use of noisy reads its totally discouraged and in page 20 right after the description of QV they claim the QV can be overestimated due to the excess of error k-mer in low quality reads. If you go the documentation the just recommend using PE data, either from WGS experiments or from 10x Linked-reads ( https://github.com/marbl/merqury/wiki/1.-Prepare-meryl-dbs) Could you prove me wrong on this?

7. An alternative way of estimating the QV of a de novo assembly would be comparing the "de novo contigs" against a high-quality reference (e.g. GRCh38). Have you tried this approach?

8. At line 449-450 you warn about the different sources of errors. Perhaps, thinking on de novo assembly different than human, also should include "substitutions" to account for those cases where the closest reference genome is slightly distant to the assembled genome (e.g., ≥1-15 Mya).

9. I would appreciate some help interpreting the tables. In the test_data you provided I found an example of heterozygous errors (haplotype switches ?) but looking at the output structural_error.bed I don´t understand the length calculation because if I read it like start-haplotig1,start-haplotig2 end-haplotig1,end-haplotig2 then the only length I get right is the haplotig1 length. Am I interpreting this correctly?

ctg1	222419;223036	223181;223037	24	HeterozygosisError	Size=762;620	-/-	28	28	28	m64012_190920_173625/116393886/ccs ….

ctg1	275279;275616	275639;275617	38	HeterozygosisError	Size=360;89	-/- ....

10. Please consider adding headers to structural_error.bed and a detailed description of the most relevant output files in github.

Minor Concerns (typos, …etc)

1. Typo 1: page 3,line 52 "dur" should be replaced by "due"

2. Typo2: page 9, lines 181-189 contains a duplicated paragraph. It should be removed.

3. Figures section. The Figure numbers should be added.

4. Figures section. The Figure legends should be added somewhere following the journal format.

5. Please consider using the term haplotype switches to refer to the heterozygous errors (or the cause of them).

Suggestions for improvement

1. Although your results do not show an increase of expansions with Flye 2.8.2, the latest version (2.8.3) "fixed rare artificial sequence insertions on some ONT datasets". As you use, flye on the error-correction module I recommend you upgrading to this version and avoid hidden biases.

2. In principle, your method should be able to distinguish SVs from errors by accounting for the coverage of each variant in the reads. Have you considered outputting the list of putative true variants?

3. Currently there are other species beside human that have been sequenced with long-reads and other technologies. For example, the Vertebrate Genome Project (https://vgp.github.io/genomeark/ ) provides sets of curated and non-curated assemblies that could be used to validate Inspector, for example, by visualizing the HiC contact maps of the genome before and after the inspector correction.

**Authors Response**

**Point-by-point responses to the reviewers’ comments:**

Reviewer 1

Comment 1

Authors describe the method Inspector, a tool with two main functions: (i) assembly evaluation and (ii) correction. Both of these functions are described in depth in the paper methodology. However comparison of the performance evaluation is done only for the "assembly evaluation" module of Inspector. IMO, the title of the paper is a bit misleading since it also does polishing.

*Response: We appreciate the reviewer’s scrutiny of our work and thoughtful suggestions for improving the manuscript. Thanks for pointing out the need for comparing the error correction module with other available methods. We have performed comprehensive comparisons with several widely used polishing tools. Apologies for the confusion regarding the title. The main function of Inspector is to evaluate the long-read de novo assembly results. Error correction is an optional function to correct obvious errors detected by Inspector. It performs local correction/polishing rather than the global polishing of other tools. We would like to keep the title as is to emphasize the focus on assembly evaluation.*

Comment 2

-One of the major challenges of the assembler is resolving repeat regions. Although authors acknowledge the effect of repeats on the assembly errors. But, there is no mention of how these errors are handled either by assembly evaluation or correction modules.

*Response: Thanks for raising this concern. Repetitive sequences are major sources of assembly errors. For assembly evaluation, Inspector can identify assembly errors from repeat regions spanned by long reads. The difficulty caused by repeats in error detection is that the positions of raw signals of assembly errors usually shift left or right within repetitive regions. Inspector utilizes a density-based clustering method to group raw assembly error signals into candidate calls, which can tolerate larger shifts in repetitive regions. For repeat regions longer than the read length, some assembly errors may be missed when reads are not aligned to the correct location. Inspector can deal with most repeat regions, as over 87% of total repeat regions (cumulative length of <15kbp repeat regions / total length of all repeat regions) can be spanned by long reads in human genomes according to the repeat annotation of GRCh38. We observed enrichment of assembly errors in repetitive regions, as shown in Fig. 3, confirming that Inspector detects assembly errors within repetitive regions.*

*For error correction, Inspector collects error-supporting reads and performs local de novo assembly at each structural error region. By selecting only local reads for assembly, Inspector excludes interference from other repeat units and simplifies assembly of repeat regions. We have added descriptions of our approach for addressing repeat regions during assembly error detection and correction in the Methods section (Lines 424-426 and 495-498).*

Comment 3

-There is no comparison against state-of-the-art assembly correction/polishing methods and there is no mention of any such methods anywhere in the paper. Since the correction/polishing of the draft assemblies is an integral part of the paper, it is important to have a comprehensive comparison against the available methods.

Nanopolish, racon, medaka, Quiver and CONSENT are some of the tools that can do assembly polishing with long reads. Comparison against existing tools is needed and will greatly improve the manuscript. I also suggest that authors cite the state-of-the-art (of assembly correction) in the introduction of the paper.

Correction method links:

CONSENT: https://urldefense.com/v3/__https://doi.org/10.1038/s41598-020-80757-5__;!!NoSwA-eRAg!QMjQbsUjnEYZkkatVIdQUBqrupyw-SYJUtKkz_GIz2UkIA_EiBr3elPeLLsoIC-h$

Quiver: https://urldefense.com/v3/__https://doi.org/10.1038/nmeth.2474__;!!NoSwA-eRAg!QMjQbsUjnEYZkkatVIdQUBqrupyw-SYJUtKkz_GIz2UkIA_EiBr3elPeLFwW2n2P$ racon: https://urldefense.com/v3/__http://www.genome.org/cgi/doi/10.1101/gr.214270.116__;!!No SwA-eRAg!QMjQbsUjnEYZkkatVIdQUBqrupyw-SYJUtKkz_GIz2UkIA_EiBr3elPeLKgT5-I8$ Medaka: https://urldefense.com/v3/__https://nanoporetech.github.io/medaka/__;!!NoSwA-eRAg!QMjQbsUjnEYZkkatVIdQUBqrupyw-SYJUtKkz_GIz2UkIA_EiBr3elPeLD1z56Tl$ Nanopolish: https://urldefense.com/v3/__https://doi.org/10.1038/nmeth.3444__;!!NoSwA-eRAg!QMjQbsUjnEYZkkatVIdQUBqrupyw-SYJUtKkz_GIz2UkIA_EiBr3elPeLI6pMMNK$

*Response: Thanks for the suggestion and information on other tools. We have tested six state-of-the-art polishing methods, including Racon, Pilon, GCpp, Medaka, Nanopolish, and CONSENT, on the HG002 assembly results of all tested assemblers (Canu, Flye, wtdbg2, hifiasm, and Shasta) based on CLR, HiFi, and Oxford Nanopore data when applicable for comprehensive comparison. Quiver is designed for the legacy PacBio RS II platform and is thus not applicable to our HG002 data generated from PacBio Sequel II. Instead, we tested GCpp, which uses the Arrow algorithm in multimolecular consensus setting and is the most recent genome polishing tool from Pacific Biosciences. We completed polishing the whole-genome assembly with Racon, Pilon, GCpp, and Medaka. However, Nanopolish and CONSENT required much longer runtimes. We tested under a variety of parameter and resource settings. However, we could not finish the task of polishing one whole-genome sample within the maximal allowed wall-clock time of our high-performance computing (HPC) server. Therefore, we selected one large contig (10Mbp in length) from each assembly result and benchmarked Nanopolish and CONSENT on the selected contig.*

*For polishing with HiFi reads, we compared Inspector to Racon, Pilon and GCPP, as they can accept HiFi data as input. Inspector corrected the most structural errors in CLR and HiFi assemblies, while Racon and Pilon notably introduced more structural errors during polishing. The four polishing methods showed similar performance in Nanopore assemblies. For small-scale errors, Inspector, Racon, and GCpp showed similar performance and corrected more errors than Pilon. We have updated Fig. 4 in the revised manuscript accordingly. A copy of the figure is also included in this response.*

*After estimating QV scores of polished assemblies (Fig. S16a), we found that Inspector achieved the greatest improvement of QV score in CLR and HiFi assemblies, while Racon achieved the largest improvement in Nanopore assembly. The QV scores were consistent with the number of assembly errors. As a comparison, we also calculated the QV scores using Merqury. All QV scores are consistent between Inspector and Merqury except the QV scores estimated by Merqury showed that Racon achieved higher accuracy than Inspector in CLR assembly (Fig. S16b), possibly because Merqury gave more weight to small-scale errors than structural errors, as it used a k-mer based approach.*

*Figure 4 Improved assembly accuracy after error correction. a Methods of assembly error correction for small-scale error and structural error. b,c Number of corrected structural (b) and small-scale error (c) in HG002 assembly. Negative values indicate more assembly errors after polishing process.*

*Figure S16 QV score improvement after polishing with PacBio HiFi reads.*

*For polishing with CLR and Nanopore data, we assessed polishing performance in the selected 10Mbp contig from each HG002 assembly. Similar to HiFi polishing, Racon and CONSENT introduced more structural errors after polishing, while Inspector, Pilon, and Nanopolish were able to correct some structural errors with noisy long reads (Fig. S17a,b). Inspector and Pilon corrected small-scale errors with CLR data, while Racon and CONSENT generated more small-scale errors in CLR and HiFi assemblies (Fig. S17c). Polishing with Nanopore reads introduced more small-scale errors into CLR and HiFi assemblies by most polishing methods (Fig. S17d), suggesting that Nanopore reads should not be used for polishing accurate assemblies.*

*In our revised manuscript, assembly polishing results are shown in Fig. 4, Fig. S16, and Fig. S17 and are discussed in the ‘Assembly error correction’ section in detail (Lines 289-313). The methods of genome polishing and options for each polishing tool are described in Methods in the ‘Polishing of HG002 assemblies’ section (Lines 602-621).*

*We have also introduced and cited the polishing methods in the introduction section (Lines 78-86).*

*Figure S17 Assembly error correction with CLR and Nanopore data. a,b Number of corrected structural errors after polishing with CLR (a) and Nanopore (b) data. Inspector fixed most structural errors among tested polishing methods in 9 and 4 out of 11 assemblies in CLR and Nanopore data, respectively. c,d Number of corrected small-scale errors after polishing with CLR (c) and Nanopore (d) data.*

Comment 4

-P8.21.Table2: Caption lists the columns in the table. But, it is difficult to follow which description belongs to which column.

*Response: Thanks for highlighting the need for greater clarity in Table 2. We have revised the legend of Table 2 to make the description of each column clearer.*

Comment 5

-P12.44: "Patterns" on different datasets should be elaborated.

*Response: Thanks for the suggestion. We have added the descriptions about repeat enrichment patterns of different datasets in Lines 266-272.*

Comment 6

-P18.19: The rationale behind using binomial distribution to correct small-scale errors should be explained.

*Response: Thanks for pointing this out. When scanning through read pileup results, Inspector first looks for candidate positions with at least 20% of reads containing mismatch/indel (to exclude most noise caused by sequencing errors and reduce computational burden). It then collects bases from all aligned reads at the candidate position to perform the binomial test. Each read can be considered as an independent experiment with two possible outcomes: having the same base with the contig or having a mismatch/indel. In theory, for each read, the probability of a mismatch/indel is 0.5 for genetic variants and 1.0 for small-scale errors, as shown in Fig. S9. As bases follow the binomial distribution, we use the binomial test to identify small-scale errors, with the null hypothesis that the probability is 0.5 (which means that the position is a genetic variant). The null hypothesis would be rejected if the p-value is significant, and a small-scale error will then be reported at this position.*

*For the error correction of small-scale errors, Inspector reports the base supported by the most reads at each error location during the error detection process. When correcting these errors, it replaces the original erroneous base with the correct base to make the contig sequence consistent with the reads. We have added a detailed description to explain why the binomial test is used for small-scale error detection in the Methods section (Lines 455-465).*

Comment 7

- regarding the previous point, inspector should be able to evaluate the assembly of species without reference genome. Now, there is an assumption that genome will be diploid which might not be correct.

*Response: Thanks for raising this issue. For polyploid species, phasing and de novo assembly is a challenging problem per se. To our knowledge, without specific experimentation (e.g., Strand-seq) prior to sequencing, there is no long-read assembler that can accurately support or solve this issue computationally. The current version of Inspector can only be applied on species with haploid or diploid genomes. For polyploid genomes, Inspector’s evaluation may be less accurate. We have emphasized this limitation in the manuscript and in the Discussion section (Lines 88, 375-383). We expect to expand the applicability of Inspector to higher ploidy levels in future versions by modeling assembly error and genetic variants in polyploid genomes.*

Comment 8

-Inspector runs in the CodeOcean capsule as described in the paper. A short summary of the experiment, describing the input dataset and outputs, in the CodeOcean may help the evaluation. Although we understand that CodeOcean is in testing as of yet.

*Response: Thanks for the advice. We have added descriptions about the input testing dataset and the primary output files for Inspector in CodeOcean.*

Reviewer 2

The method addresses the lack of tools to evaluate the accuracy in genome assembly using long reads (LR) and without reference. The authors propose a simple yet effective way of identifying potential errors in de novo genome assemblies.

Comment 1

I have a few comments for the authors to consider:

- One paragraph is duplicated (lines 181 - 189)

*Response: Thanks for noticing this mistake. We have removed the duplicated paragraph.*

Comment 2

- Inspector is designed to work on LR-only datasets, with most success on HIFI data. Have the users tried to assess how much gain, if any, would there be if an additional Illumina short-read dataset is available? Like the authors have said, it is becoming the norm for people to use HIFI-only data for assembly. However, it would be good to see hard evidence once and for all to make an informed decision that the magnitude of gain is not worth the investment to produce short-read datasets on top of LR, or not.

*Response: Thanks for the suggestion. To estimate the gain from an additional short-read dataset, we used an Illumina dataset to perform short-read polishing with Racon and Pilon on HG002 assemblies and evaluated assembly quality before and after short-read polishing. We found that the additional short-read dataset improved the accuracy of CLR and Nanopore assemblies mainly by correcting small-scale errors. However, the quality of HiFi assemblies did not show significant improvement, as the HiFi reads already have high base accuracy and there were not many small-scale errors present in HiFi assemblies. More structural errors were introduced into the assembly after polishing with Racon (9 out of 11 assemblies) and Pilon (8 out of 11 assemblies), suggesting that a short-read dataset is not suitable for fixing structural errors due to limited read length. The results regarding short-read polishing are shown in Fig. S18 and discussed in the ‘Assembly error correction’ section (Lines 314-320). As expected, the results suggest that accurate short reads can fix base errors for noisy long reads and thus improve assembly quality. However, if we already have HiFi reads, it seems unnecessary to generate additional short-read data.*

*Figure S18 Polishing HG002 assemblies with Illumina dataset*

Comment 3

- LR de novo genome assembly is gaining a lot of tractions in discovering and studying plants and animals. Have the authors assessed the performance of Inspector on polypoidy genomes and other "difficult" genomes? If Inspector is designed to work only on haploids and diploids (which would limit its potential, in my opinion), it should have been made clearer.

*Response: Thanks for pointing out the need to discuss usefulness in polyploid genomes. At this stage, Inspector works only on haploid and diploid genomes. It identifies small-scale errors with the binomial test, with the assumption that the genome is diploid. For polyploid genomes, it is more challenging to distinguish true assembly errors from genetic variants. We plan to train models for genetic variants and assembly errors in polyploid genomes and expand the application of Inspector to higher ploidy levels in the future versions. We have stated this more clearly in Line 88 and in the Discussion section (Lines 375-383).*

Comment 4

- Would the authors consider comparing with other common polishing tools (e.g. Pilon, Arrow, Racon,....) and assess performance of individual tools and/or combos of tools in correcting assembly errors?

*Response: Thanks for the suggestion to compare with other tools. We have tested six commonly used polishing methods on the HG002 assemblies for comparison These methods are Racon, Pilon, GCpp, Medaka, Nanopolish, and CONSENT. For polishing with HiFi reads, we compared Inspector to Racon, Pilon and GCpp, as they can accept HiFi data as input. As shown in Fig.4, Inspector fixed the most structural errors in CLR and HiFi assemblies, while Racon and Pilon actually introduced more structural errors after polishing. The four polishing methods showed similar performance in Nanopore assemblies. For small-scale errors, Inspector, Racon and GCpp showed similar performance and corrected more errors than Pilon.*

*Figure 4 Improved assembly accuracy after error correction. a Methods of assembly error correction for small-scale and structural errors. b,c Number of corrected structural (b) and small-scale errors (c) in HG002 assembly. Negative values indicate more assembly errors after the polishing process.*

*After estimating QV score of polished assemblies (Fig. S16a), we found that Inspector achieved the greatest improvement of QV score in CLR and HiFi assemblies, while Racon achieved the largest improvement in Nanopore assemblies. The QV scores were consistent with the number of assembly errors. QV scores estimated by Merqury showed that Racon achieved the highest accuracy in CLR assembly than Inspector (Fig. S16b), possibly because it gave more weight to small-scale errors than structural errors as it used a k-mer based approach.*

*Figure S16 QV score improvement after polishing with PacBio HiFi reads.*

*We have also assessed the polishing performance of different polishing tools with CLR and Nanopore reads. Racon, Pilon, and CONSENT were tested with CLR data. Racon, Pilon, Medaka, and Nanopolish were tested with Nanopore data. The results are shown in Fig. S17.*

*The comparison to other polishing tools are discussed in the ‘Assembly error correction’ section (Lines 289-313).*

*Figure S17 Assembly error correction with CLR and Nanopore data*

Comment 5

- Table 1: have you analyzed the characteristics of Inspector's false negatives? Why and how are they missed? e.g. Authors commenting that is "possibly because of the predominance of indel", but obviously Inspector can deal with some indels but not the others. Perhaps they can look at whether the FNs are all sporadic or there are some characteristics that would allow them to be caught, or are there any further explanations as to why they are missed (e.g. missing those are particularly long in length? particular difficulty around centromere?)

*Response: Thanks for the question about false negatives. Inspector achieved high accuracy in detecting structural errors (F1-score 97-99%). The detection of small-scale errors was also high with HiFi reads (F1-score over 99%) but lower with CLR reads (F1-score 91-92%). We have checked the accuracy of detecting each subtype of small-scale errors and found that most false negatives were due to small-expansions and small-collapses. As shown in Table R1, the sensitivities of small expansions and small collapses with a CLR dataset were lower than the HiFi dataset in both haploid and diploid simulations.*

*We further investigated the false negatives in CLR evaluation. The proportion of reads supporting assembly errors was less in false-negative than in true-positive events (Fig. R1a). These false negatives were missed because there were fewer reads supporting assembly errors, indicating that the binomial test would not be significant, and these positions would be filtered out from the assembly error list. The sensitivity was higher in HiFi datasets as there were not many low-ratio events (Fig. R1b), which means the lower ratio in the CLR dataset was due to the presence of sequencing errors.*

*We then checked the proportion of error-supporting read for each subtype of small-scale errors and found that most low-ratio false-negative events were small expansions and small collapses (Fig. R1c). The predominant type of sequencing error is indel (small insertions). Small collapses and small expansions were more susceptible to indel-like sequencing errors, as the presence of indel-like sequencing errors may alter local read alignment, make the read NOT supporting the assembly error, and cause a lower error-supporting read ratio (Fig. R1d,e). These false-negatives can be detected if we loosen the threshold for the p-value of the binomial test and allow lower-ratio events to be reported as assembly errors. However, this adjustment will result in more false positives caused by genetic variants and eventually worsen accuracy in detecting assembly errors. To maintain higher confidence in reporting assembly errors, we have kept the current threshold and require the binomial test to be significant when reporting a small-scale error. We have updated Fig. S4 and described missed events in the ‘Benchmark with simulation’ section (Lines 138-142).*

*Figure R1 small-scale error detection in simulated assembly. a,b Distribution of error-supporting read at small-scale error positions from CLR (a) and HiFi (b) dataset. c Distribution of error-supporting read for three subtypes of small-scale errors. d,e Examples of small expansion (d) and small collapse (e) missed by Inspector because of indel sequencing errors.*

Comment 6

- Table 1: similar to above, what are the assembly errors that are missed by Merqury but not Insepctor?

*Response: Thanks for this question as well. We have characterized assembly errors detected by Inspector but not Merqury in both haploid and diploid simulated datasets. We first checked the subtype and found that approximately 54% of these assembly errors were small collapses (Fig. S5a). As we simulated 50% base substitutions, 25% small collapses and 25% small expansions, there is strong enrichment of small collapses in Merqury-missed events. These findings suggest that Merqury has less power for detecting small collapses.*

*We then computed the coordinates of these assembly errors on the reference genome and annotated them using the same method that we used to annotate assembly errors in HG002. We found that approximately 70.4% of Merqury-missed assembly errors were located in repetitive regions (Fig. S5b). It is more difficult to detect assembly errors in repetitive regions using a k-mer based approach, as k-mers in repetitive regions are typically duplicated and occur multiple times in the assembly. In these cases, Inspector can still identify these events, as it detects assembly errors from long-read alignments, which have better potential to be aligned properly to repetitive regions using parts of unique sequences in long reads. We have added Fig. S5 and descriptions of Merqury-missed assembly errors to the ‘Benchmark with simulation’ section (Lines 145-147).*

*Figure S5 Small-scale error missed by Merqury but detected by Inspector in the simulated dataset. a Three subtypes of small-scale errors detected by Inspector but not by Merqury in haploid (left) and diploid (right) simulation. b Composition of Merqury-missed assembly errors located within and outside the repetitive regions for haploid (top) and diploid (bottom)*

Comment 7

- Line 210: I might have missed it but I can't find detailed descriptions of what filters are applied on structural errors. For small-scale errors, the binomial test is clear. Is it possible to apply an appropriate statistical test for the structural errors as well, instead of an arbitrary filter which can be hard to judge what threshold one should use?

*Response: Thanks for raising this. Unlike small-scale errors, structural errors usually span a larger region. For small-scale errors, reads follow the binomial distribution, as one read can either support or not support an error. However, for structural errors, the read alignments are more complex. There are more possible outcomes for reads (such as errors involving different types, sizes, and positions). It is also difficult to obtain the total number of reads, as some reads may partially cover the assembly error region. We have found it challenging to fit to regular statistical test and decided to filter the candidates based on the features such as ratio of error-supporting reads, local coverage, and mapping quality.*

*The most important criterion in Inspector’s current filter is the ratio of error-supporting reads. This ratio is computed by the number of error-supporting reads divided by the local coverage (estimated with coverage of flanking regions on both sides). It is the fundamental difference that distinguishes structural errors from heterozygous SVs, as shown in Fig. S9. The default threshold was modeled on both simulated and read datasets for different types of long reads. We suggest that users specify the input data type (CLR, HiFi or Nanopore) and use the default threshold, as it will generate more stable outcomes. We have added descriptions of structural error filtering in the Methods section in Lines 431-443.*

Comment 8

- Any comment on actual run time and hardware requirement?

*Response: Thanks for asking computational performance and requirements. Yes, we have assessed runtime and memory usage for Inspector and other evaluation or polishing methods tested in this manuscript. We have added two supplementary tables and a paragraph in the Results section (Lines 333-337) to provide runtime and memory usage for assembly evaluation and error correction. The requirements for Inspector are moderate and should be easily achievable for most users. We have also included comments about required computational resources in the Discussion section (Lines 351-362).*

Reviewer 3

Dear Chen et al,

This is a good work and the manuscript is really well written. I think Inspector could be a very valuable tool for miss-assembly detection in de novo assembled genomes. The program is easy to install following the github link provided. In principle, could be used to distinguish between true variants and error. This would be particularly interesting to detect regions that are heterozygous for (SVs) in de novo assembled genomes. Thus, opening the door to study their biological implications. Your team made a clever use of the binomial distribution to both detect errors (at small and large scale) and apply these methods to identify errors in different assemblies obtained with long-reads of decreasing error rate (nanopore, pacbio CLR and Hifi reads). In addition, the results on the human (HG002) datasets are promising. However, I still have some concerns about the reliability of the methods used, the interpretation of the results and the application of the tool to improve newly assembled genomes. For these reasons I would reject the present version of the manuscript until you address my main concerns. I will be happy to review it again after you revise the current version of the manuscript. I hope the comments below help to strengthen Inspector and improve its applicability.

Sincerely,

Anonymous reviewer

*Response: Thanks for the positive comments about our work, which is really encouraging. We have addressed the reviewer’s concerns by performing additional experiments. These comments are very helpful for the improvement of Inspector and the manuscript.*

Major Concerns

1.	The estimation of Quality Value scores shown here infers the error rate from noisy reads to contigs from long-read only assemblies. In my opinion the estimate error rate is a mixture of both the errors coming from the reads and the assemblies. I think the right way to do this would be using high-accuracy reads against the assemblies (e.g. Merqury paper). Could you explain why you decided to do so?

*Response: Thanks for your question about the error rate. Inspector estimates Quality Value score using only errors from assemblies. It can exclude errors from reads (sequencing errors) during the assembly error detection process.*

*Merqury uses a k-mer based approach for identifying assembly errors. Both contigs and reads are cut into smaller k-mers (21bp for human genome by default), and the occurrence of the k-mer in all reads is used to determine if this k-mer contains an assembly error. Even a single sequencing error within 21bp will cause a missing count for the k-mer occurrence. Since Merqury requires perfect matching between k-mers in contigs and k-mers in reads, the base accuracy of reads largely affects the evaluation process. Thus, high accuracy reads (e.g., Illumina reads) are required for Merqury’s evaluation.*

*Unlike Merqury, Inspector identifies assembly errors based on read alignments. When Inspector scans through the contigs, it stacks up aligned reads and counts how many reads have different bases against the contig (potential assembly error). For example, in Fig. R2, PacBio CLR reads were aligned to the assembly, and all sequencing errors were randomly distributed within read alignments. When there is an assembly error (at position contig_1060:615189-615189), 83% of aligned reads have different bases against the contig. At other positions, fewer than 20% of reads contain errors, because sequencing error rate for noisy long reads ranges between 5% and 20%. Inspector performs the binomial test to report an assembly error only when more than 50% of reads differ from the contig, eliminating noise from sequencing errors. As shown in Fig. R2, within this 100bp window, only one assembly error of 1bp is reported and used for QV estimation.*

*Figure R2 Distinguish assembly error from noisy long-read alignment.*

*High-accuracy reads simplify the assembly error detection process, and we observed the highest error-detection accuracy with a PacBio HiFi dataset, as shown in Table 1. However, Inspector also achieved high precision with PacBio CLR reads (sequencing error rate of ~15%), indicating that Inspector is capable of reporting errors merely from assembly results, even with noisy reads as input. Given high coverage (30X and higher), detection of assembly error and estimation of quality value can tolerate the presence of sequencing errors.*

*In addition, due to advances in long-read de novo assembly algorithms, long-read-only de novo assembly is becoming routine. There is no need to generate another set of high-coverage short-read data, and our evaluations demonstrate that short reads do not provide significant gain in improving assembly accuracy. Our findings highlight Inspector’s intended value for long-read de novo assembly evaluation. Specifically, besides small-scale errors, Inspector can accurately evaluate structural errors, thus providing functionality that is not available for the community.*

*We have emphasized these points in the Methods section of our revised manuscript.*

2.	The QV scores are higher at low coverage (Figure S11 c), what is the reason for this?

It looks like with lower coverage you are just having less power to detect enough errors in the assemblies. Your equation in fact does not weight the estimates by depth. Therefore, at 10x you detected less errors and the QV looks higher than expected. In my opinion a way to overcome this would be to divide the errors by the total number of bases covered above certain threshold depth (≥ 5x). Uncovered bases and Ns should not be considered in the denominator (p.9). Do you agree?

*Response: Thanks for pointing out this issue and for the excellent suggestions. The purpose of this down-sampling experiment was to determine how much coverage is required for accurate assembly evaluation with Inspector. It is suggested that users use datasets with at least 30x to obtain accurate assembly error lists, especially for structural errors.*

*It is true that assembly error detection is more difficult at lower coverage (10x and 20x), as there are too few reads to distinguish true errors from genetic variants or sequencing errors. The QV score was initially estimated based on identified assembly errors; thus, it was higher when fewer errors were reported at 10x.*

*As suggested, we have modified code in Inspector to exclude N bases and bases with too low or too high coverage. We also lowered the p-value cutoff for low-depth datasets to rescue missed small-scale errors due to insufficient depth. The updated results are shown in Fig. S12 (original Fig. S11). After updating the QV calculation and small-scale error detection methods, we observed similar numbers of small-scale errors reported at different sequencing depths (Fig. S12a), and the QV score was consistent even at lower depths (Fig. S12b). Assembly using hifiasm showed larger fluctuations because the error rate for hifiasm was too low (0.001%), and some small differences in numbers of assembly errors can cause large differences in QV scores.*

*We have also updated the results with QV scores (Table 2, Fig. 4, Fig. S6, and Fig. S12) and the QV estimation description in the Methods section. Most QV scores were slightly lower than before, as some uncovered bases were excluded, and the error rate was therefore slightly higher. We have also submitted the latest version of Inspector to GitHub and CodeOcean.*

*Figure S12 Inspector evaluation with down-sampled dataset.*

3.	Figure S11 b shows a higher number of Structural errors produced with Flye 2.8.2 using HiFi reads. Do you have any explanation for this?

*Response: Thanks for this question. In Fig. S11b, there are more structural errors with Flye assembly when the numbers are stabilized after 30x. We further investigated four subtypes of structural errors, as shown in Fig. R3. The structural errors of Flye assembly consisted of expansions, collapses, haplotype switches (Heterozygosis errors in old terms), and inversions. Although the numbers of expansions and collapses were low, there were many more haplotype switches contained in Flye assembly than with the other three assemblers. This difference indicates that there may be systematic error when Flye processes bubbles at heterozygous regions. This result is also consistent with our results shown in Fig. 2, in which Flye showed a dominance of haplotype switches in all CLR, HiFi, and Nanopore assemblies and more haplotype switches than other assemblers in both CLR and HiFi assemblies.*

*Figure R3 Four types of structural error.*

4.	Shasta normally to produce shorter assemblies than all the other assemblers, however the amount of collapse errors using nanopore is a lot lower than for wtbg Are the collapse errors detected in Shasta assemblies spanning larger regions? What is the cumulative length of the collapsed errors compared to other assemblers?

*Response: Thanks for pointing out this issue with collapse errors and the suggestions for further investigation. We have checked the collapses in the four Nanopore assemblies of HG002. As shown in Table R2, there were 227 collapses in the Shasta assembly with a median length of 104bp. As suggested, we also calculated the cumulative length of the collapse errors, but the cumulative length of these collapse errors alone cannot explain Shasta’s shorter assemblies (Table R2).*

*We further evaluated the completeness of individual assembly results by mapping the assemblies to the reference genome. In Table R2, we show the mapping rate of assembly, coverage of reference genome, and percentage of reference genome covered with depth of 0,1,2 or higher. With the highest number of total bases, the mapping rate as well as the coverage of wtdbg2 assembly was lower than with the other three assemblers. Shasta had similar mapping rates to Canu and Flye. However, Shasta has the lowest genome coverage among the three results. This difference can explain why Shasta assembly resulted in the fewest total bases. When we stratified the depths into 0, 1, 2, and >2, we found that Shasta had more uncovered bases (depth=0) and less properly covered bases (depth=1) than Canu and Flye. In addition, both Canu and Flye can perform haplotype-resolved de novo assembly that generates contigs for both haplotypes at heterozygous regions in theory. We observed more regions covered by >1 contigs (depths of 2 and higher) in Canu and Flye than in Shasta, which may be another reason why Shasta assembly is shorter in length.*

5.	Again, about the QV estimates. In the methods you wrote that you used Merqury (Rhie et al. 2020) and you built meryl databases from a fasta. Was this a "reads" fasta or a reference genome (GRCh58)?, what have you used?

*Response: Thanks for this question. The FASTA file was reads. For the simulated dataset, the simulated PacBio HiFi reads (base accuracy > 98%) were used to generate a meryl database for Merqury, as there was no short-read dataset available. For the HG002 assembly evaluation, we used a paired-end Illumina dataset to generate a meryl database. We have revised the Merqury evaluation section in the Methods to improve clearity (Lines 535-538, 563-565).*

6.	If you used reads, the method is meant to use high-accuracy reads at enough coverage to estimate the sequence accuracy in the assembly. The use of noisy reads its totally discouraged and in page 20 right after the description of QV they claim the QV can be overestimated due to the excess of error k-mer in low quality reads. If you go the documentation the just recommend using PE data, either from WGS experiments or from 10x Linked-reads ( https://urldefense.com/v3/__https://github.com/marbl/merqury/wiki/1.-Prepare-meryl-dbs__;!!NoSwA-eRAg!QMjQbsUjnEYZkkatVIdQUBqrupyw-SYJUtKkz_GIz2UkIA_EiBr3elPeLLheuPiw$ ) Could you prove me wrong on this?

*Response: Thanks for highlighting this issue. The reviewer is correct. For the benchmark of Merqury, we provided high-accuracy reads to Merqury for both simulated and HG002 assembly evaluation. For the simulation, a simulated PacBio HiFi dataset with depth of approximately 50X was adopted. For HG002 assembly evaluation, we built the meryl database from a 50X paired-end (2x150bp) Illumina WGS dataset. We have revised the Methods section and clarified which datasets were used for Merqury (Lines 535-538, 563-565).*

7.	An alternative way of estimating the QV of a de novo assembly would be comparing the "de novo contigs" against a high-quality reference (e.g. GRCh38). Have you tried this approach?

*Response: Thanks for this suggestion. Yes, Inspector includes a reference-based mode that evaluates the assembly by comparing assembled contigs to the reference genome. We used this reference-based mode to assess how complete and redundant the assembly was (reference-based mode in Table 2) and to visualize potential ultra-large structural errors (Fig. S8). However, estimating QV (and detecting assembly errors) based on the reference genome is not the most accurate approach because of the diversity between sequenced and the reference genome. When we observe difference between an assembly and the reference genome, this difference may either be a true assembly error or a genetic variant from the sequenced sample. It is impossible to distinguish them simply based on the contig-to-reference comparison. If we consider all differences as assembly errors, the QV score will be tremendously underestimated. Human genome contains ~0.1% single nucleotide variants, resulting in QV scores that are always lower than 3, in theory. Therefore, Inspector utilizes the reference genome to evaluate the assembly at a larger scale and uses reads from the same sequenced subject to estimate QV more accurately.*

8.	At line 449-450 you warn about the different sources of errors. Perhaps, thinking on de novo assembly different than human, also should include "substitutions" to account for those cases where the closest reference genome is slightly distant to the assembled genome (e.g., ≥1-15 Mya).

*Response: Thanks for the suggestion. We have added “substitutions” as a potential cause of assembly errors in the Methods section.*

9.	I would appreciate some help interpreting the tables. In the test_data you provided I found an example of heterozygous errors (haplotype switches ?) but looking at the output structural_error.bed I don´t understand the length calculation because if I read it like start-haplotig1,start-haplotig2 end-haplotig1,end-haplotig2 then the only length I get

right is the haplotig1 length. Am I interpreting this correctly?

ctg1 222419;223036 223181;223037 24 HeterozygosisError Size=762;620 -

/- 28 28 28 m64012_190920_173625/116393886/ccs ....

ctg1 275279;275616 275639;275617 38 HeterozygosisError Size=360;89 -/- ....

*Response: Thanks for alerting us about this issue. Yes, the reviewer was correct. The second and third columns are the start and end locations on haplotig1 and haplotig2. In haplotype switches, we observed expansion-like patterns in one haplotig and collapse-like patterns in the other haplotig (shown in Fig. S2c). By default, Inspector selects ‘Expansion’ in haplotig1 and ‘Collapse’ in haplotig2. The ‘Expansion’ on haplotig1 indicates that some contig sequences are extra, and thus the Size equals END-START on haplotig1. On haplotig2, a ‘Collapse’ indicates some sequence is missing at this location, so the END position is only 1bp after the START position, and the true lengths of the missing sequences are represented by Size in sixth column. We have added a detailed description to our README file on the GitHub page.*

10.	Please consider adding headers to structural_error.bed and a detailed description of the most relevant output files in github.

*Response: Thanks for the suggestion for improving clarity. We have modified the source code to add header lines to both structural and small-scale error lists. We have also added a detailed description to the GitHub page under the section ‘Output file descriptions’ to explain what each column represents.*

Minor Concerns (typos, ...etc)

1.	Typo 1: page 3,line 52 "dur" should be replaced by "due"

2.	Typo2: page 9, lines 181-189 contains a duplicated paragraph. It should be removed.

3.	Figures section. The Figure numbers should be added.

4.	Figures section. The Figure legends should be added somewhere following the journal format.

5.	Please consider using the term haplotype switches to refer to the heterozygous errors (or the cause of them).

*Response: Thanks very much for the careful review.*

*1.	We have corrected this typo.*

*2.	The duplicated paragraph has been removed.*

*3.	We have added figure numbers to each figure page.*

*4.	As instructed by the journal format and example papers, we have put Figure Legends to the end of the main text.*

*5.	Nice suggestion! We have changed the “heterozygosis error” to the term “haplotype switch” in the revised version and in the output files of Inspector.*

Suggestions for improvement

1.	Although your results do not show an increase of expansions with Flye 2.8.2, the latest version (2.8.3) "fixed rare artificial sequence insertions on some ONT datasets". As you use, flye on the error-correction module I recommend you upgrading to this version and avoid hidden biases.

*Response: Thanks for the note. In the revised version, we have upgraded Flye to version 2.8.3 and repeated all error correction experiments with CLR, HiFi, and Nanopore data on HG002 assemblies. We observed higher structural error-correction rate after upgrading to v2.8.3 overall, due to a higher rate of successful local assembly with Flye (generated full-length contig that can be aligned back to the same error-flanking region). The results in Fig. 4, Fig. S17, Table S3, and Table S4 have been updated using Flye 2.8.3. We have also updated the documentation in GitHub page regarding Flye version.*

2.	In principle, your method should be able to distinguish SVs from errors by accounting for the coverage of each variant in the reads. Have you considered outputting the list of putative true variants?

*Response: Thanks for the suggestion. We have considered SV detection, as the false-positive assembly errors filtered out by Inspector are the genetic variants anyway. However, Inspector can only report assembly errors and heterozygous SVs, but not homozygous SVs. Inspector distinguishes assembly errors from SVs based on read-to-contig alignment. As shown in Fig. S9, homozygous SVs have both haplotypes identical with the contig and 0% of the reads contain differences against the contig. When Inspector scans through read alignments, there is no signal at all in homozygous SV regions, thus no SV events can be reported. If we output the SV list, it will contain only heterozygous events. In addition, coordinates reported by Inspector are based on the contig (e.g., Contig_1:5000-6000) instead of the reference genome (e.g., chr1:10000-11000). To generate an SV callset, we would need to map coordinates from the contigs with respect to the reference genome. This process cannot be generalized for genomes without a reference genome.*

7.	Currently there are other species beside human that have been sequenced with long- reads and other technologies. For example, the Vertebrate Genome Project (https://urldefense.com/v3/__https://vgp.github.io/genomeark/__;!!NoSwA-eRAg!QMjQbsUjnEYZkkatVIdQUBqrupyw-SYJUtKkz_GIz2UkIA_EiBr3elPeLFpp0g46$ ) provides sets of curated and non-curated assemblies that could be used to validate Inspector, for example, by visualizing the HiC contact maps of the genome before and after the inspector correction.

*Response: Thanks for the nice suggestion. We obtained the long-read sequencing data (PacBio CLR) of Anna’s Hummingbird from the Vertebrate Genome Project (VGP) and performed whole-genome assembly with Canu, Flye, and wtdbg2. We used the same CLR dataset to correct assembly errors identified by Inspector in these three assemblies. As the manually curated genome sequence of this Anna’s Hummingbird sample is available from VGP, we directly aligned the uncorrected and Inspector-corrected assemblies to the curated genome to visualize the structural error correction. The error correction validation in the Anna’s Hummingbird genome is shown in Fig. S19-S20 and discussed in the ‘Assembly error correction’ section*

*(Lines 321-330). We also added the methods for assembly and error correction in Methods section (Lines 623-630).*

*Figure S19 Inspector error correction in Anna’s Hummingbird genome assemblies*

*Figure S20 Example of structural errors corrected by Inspector error-correction module*

**Second round of review**

**Reviewer 2**

The authors have provided detailed responses to address all of the points I raised.

**Reviewer 3**

Dear Chen et al,

First of all, congratulations for the work done. I am very glad of your thorough revision of the manuscript. You have actually addressed all reviewer’s comments including mine. I really appreciate the description of the new out files with headers and the error-correction evaluation on the hummingbird genome. However, I still have some doubts, concerns and suggestions to improve the manuscript before its final publication.

Sincerely,

Reviewer3

Minor Concerns

Related to your answers

1. I am more convinced about the estimation of Quality Value scores based on assembly errors and how you distinguish them from sequencing errors. It seems more stable and “robust” to coverage fluctuations. However, I think is still a proxy for the QV because it compares the assembled reads used as input with the final output (assembled contigs). There’s obviously a background of sequencing errors in less than 50% of the reads that can contribute to a low base-accuracy of the assembly. In fact, you observed a reduction of small-scale errors with illumina polishing (Gcpp and Pilon, Fig 4c) that should be taken into consideration. I would call your measure long-read QV or QV proxy, as there are low-quality regions where you don’t detect errors but are less accurate. These regions are covered go into the denominator and therefore reduce the error rate, artificially increasing the QV. This will help distinguishing form the merqury estimate. I think it would be anyway very useful for evaluating long-read assemblies.

2. I agree on avoiding the use of a reference genome from a different individual for QV estimation due to the heterozygosity. However, if we consider rate of polymorphism as the error rate (0.1%), in the case of the human genome QV≤30 (Q=10*log10(0.001)). Thus, for highly homozygous species could be an alternative approach.

3. Regarding to SV error and true SV calling in one go: I understand is not the objective of Inspector to report variants but to report true assembly errors. However, from one sample the only structural variants detectable should have a heterozygous genotype in the assembled sample. To really identify regions with real “homozygous” SVs one should screen several samples and map everything to the same reference.

4. Inspector was designed to work with contigs, so it should be run on contigs before any scaffolding. However, is still a valuable tool to detect how consistent is the final assembly with the long-read data after scaffolding.

5. The results shown in Fig19 are convincing. FigS20 shows a cherry-picked example. That’s fine but you should indicate the exact coordinates (contig and position) in the Hummingbird assembly. Also, you need to provide the exact assembly version used to evaluate the error-correction module. Was an assembly broken into contigs or you used the final curated scaffolds?

Regarding to the updated version of the program:

1. Last version and detailed description is under ChongLab but the actual address is https://github.com/Maggi-Chen/Inspector (double-check line 666)

2. You repeatedly wrote that inspector generates evaluation plots (line 97-98). However, it seems that they are only done in the reference base mode. I think some of them as the N plots could be done in absence of a reference …don´t you think?

3. The updated version is supposed to work well with Flye 2.8.3, and you said you have tested it with a reduction on assembly errors. However in the main text still says Flye 2.8.2 (line 546) likely to avoid redoing all the analyses, that's understandable. But where are the results comparing the Flye 2.8.2 and 2.8.3 evaluation?

4. With this version the error correction was run on the testdata producing the error below. But this error did not happened with previous version: Inspector-correct.py -i check_inspector_review1/ --datatype pacbio-raw -o check_inspector_review1/ &> assembly_correction.log

(inspector_review1) [reviewer3@cnb5 check_20210915]$ more assembly_correction.log

TIME for validating parameter 0.00145697593689

TIME for reading contig and length 0.00900793075562

TIME for reading assembly errors 0.0235669612885

Base error correction for ctg1 finished. Time cost: 0.00782895088196

Traceback (most recent call last):

File "/home/devel/reviewer3/bin/programs/Inspector_review1/Inspector/inspector-correct.py", line 104, in

inspector_correct.error_correction_large(chrominfo,ctginfo[chrominfo],aectg[chrominfo],snpctg[chrominfo],bamfile,outpath,inscor_args.datatype,inscor_args.thread/3)

File "/home/devel/reviewer3/bin/programs/Inspector_review1/Inspector/denovo_correct.py", line 409, in error_correction_large

aeset=findpos(aeset,snpset,bamfile,outpath,datatype,thread)

File "/home/devel/reviewer3/bin/programs/Inspector_review1/Inspector/denovo_correct.py", line 105, in findpos

aestart=int(c.split('\t')[1])

ValueError: invalid literal for int() with base 10: '222419;223036'

Is this a bug?

Main Text

1. L75-77 I think you should rephrase this a bit “BUSCO…evaluates conserved genomic regions and is not informative on the most divergent sequences in the genome”

2. Figure S16b look at the axis revise the English I think “increase” or “Improvement” are correct words and “increasement” is not.

3. L. 361 typo COSNENT -> CONSENT

4. L.458 “with n bring” -> “with n being”

5. L.509-511 I think brackets will help distinguishing variants from substitutions. “…genetic variants of the sequenced genome (including SVs, SNPs and indels) and substitutions.”

**Authors Response**

**Point-by-point responses to the reviewers’ comments:**

Comment

Reviewer #2: The authors have provided detailed responses to address all of the points I raised.

*Response: We thank Reviewer 2 again for the insightful suggestions to improve the manuscript.*

Reviewer #3: Dear Chen et al,

First of all, congratulations for the work done. I am very glad of your thorough revision of the manuscript. You have actually addressed all reviewer's comments including mine. I really appreciate the description of the new out files with headers and the error-correction evaluation on the hummingbird genome. However, I still have some doubts, concerns and suggestions to improve the manuscript before its final publication.

Sincerely,

Reviewer3

*Response: We appreciate the positive comments from Reviewer #3. We are also grateful for all the suggestions and comments which are very helpful for strengthening our manuscript.*

Minor Concerns

Related to your answers

1.	I am more convinced about the estimation of Quality Value scores based on assembly errors and how you distinguish them from sequencing errors. It seems more stable and "robust" to coverage fluctuations. However, I think is still a proxy for the QV because it compares the assembled reads used as input with the final output (assembled contigs). There's obviously a background of sequencing errors in less than 50% of the reads that can contribute to a low base-accuracy of the assembly. In fact, you observed a reduction of small-scale errors with illumina polishing (Gcpp and Pilon, Fig 4c) that should be taken into consideration. I would call your measure long-read QV or QV proxy, as there are low-quality regions where you don't detect errors but are less accurate. These regions are covered go into the denominator and therefore reduce the error rate, artificially increasing the QV. This will help distinguishing form the merqury estimate. I think it would be anyway very useful for evaluating long-read assemblies.

*Response: We agree that the QV estimation is likely to be affected by small-scale errors in low-complexity regions using noisy long reads. However, the QV scores are helpful to evaluate a suite of assemblers on the same data overall. This comparison is consistent and robust – better tools will have higher QV scores. Merqury may potentially include small-scale errors with high-accuracy Illumina data by design. However, the k-mer based approach still compromises in the low-complexity regions where the assembly errors have identical sequences (k-mers) with other region(s) of the genome. In addition, Merqury needs high-accuracy data and therefore cannot perform long-read-only de novo assembly evaluation. And it cannot distinguish different types of assembly errors. The merits of Inspector are to fill in these gaps in the community.*

2.	I agree on avoiding the use of a reference genome from a different individual for QV estimation due to the heterozygosity. However, if we consider rate of polymorphism as the error rate (0.1%), in the case of the human genome QV≤30 (Q=10*log10(0.001)). Thus, for highly homozygous species could be an alternative approach.

*Response: We agree that the QV estimation with reference genome can be accurate for species with very low diversity. The reference-based mode of Inspector can use the reference as the ground-truth to directly identify assembly errors and provide a more accurate estimation of QV for such species.*

3.	Regarding to SV error and true SV calling in one go: I understand is not the objective of Inspector to report variants but to report true assembly errors. However, from one sample the only structural variants detectable should have a heterozygous genotype in the assembled sample. To really identify regions with real "homozygous" SVs one should screen several samples and map everything to the same reference.

*Response: We agree with the Reviewer’s insight on this issue, and we appreciate the suggestion to include SV output in the pipeline. We may list the putative (of course not comprehensive) SVs in future versions. This might be helpful for the estimation of some population genetic parameters or technical diagnostic applications.*

4.	Inspector was designed to work with contigs, so it should be run on contigs before any scaffolding. However, is still a valuable tool to detect how consistent is the final assembly with the long-read data after scaffolding.

*Response: It is true that Inspector is designed for assembled contigs. This is based on the fact that most of the current long-read assemblers only generate the contig sequences. Flye, for example, is one of the very few algorithms that can perform scaffolding. It attempts to order the assembled contigs based on the repeat graph structure and connect them into scaffolds with gaps represented with Ns. By default, Flye doesn’t perform scaffolding in the latest version. However, in theory, Inspector can also be applied on scaffolds. The gaps (Ns) in the scaffolds should not affect the assembly error detection process. But the continuity statistics would be largely over-estimated (largest contig length, N50, etc.), as the scaffolds are not actually fully reconstructed. Anyway, this is a very good point. We will further investigate this issue and update this function in future versions.*

5.	The results shown in Fig19 are convincing. FigS20 shows a cherry-picked example. That's fine but you should indicate the exact coordinates (contig and position) in the Hummingbird assembly. Also, you need to provide the exact assembly version used to evaluate the error-correction module. Was an assembly broken into contigs or you used the final curated scaffolds?

*Response: We thank the reviewer for the affirmation of our error-correction results. We have labeled the coordinates of assembly errors in Fig. S20 and added the version of curated Anna’s hummingbird assembly to the ‘Availability of data and materials’ section. The final curated scaffolds were used for evaluation without breaking into contigs. They served as the reference during the evaluation of the Canu, Flye, and wtdbg2 de novo assemblies.*

Regarding to the updated version of the program:

1.	Last version and detailed description is under ChongLab but the actual address is https://urldefense.com/v3/__https://github.com/Maggi-Chen/Inspector__;!!NoSwA-eRAg!XNB51dZruGPJ813V_LBsTkxn8YZS4P71y9O2zBCsG527qwJzVPzuOqoXMhPnfeLO$ ( double-check line 666)

*Response: We thank the reviewer for pointing this out. The repository under ChongLab is forked from the GitHub of Maggi-Chen (the first author) before our last revision. We have synchronized the fork in ChongLab. Now the GitHub page stated in the manuscript should be the latest version and contain detailed descriptions.*

2.	You repeatedly wrote that inspector generates evaluation plots (line 97-98). However, it seems that they are only done in the reference base mode. I think some of them as the N plots could be done in absence of a reference ...don´t you think?

*Response: We thank the reviewer for the suggestion. Inspector does generate the N plots in absence of the reference in the current version. In the test dataset on CodeOcean, we did not provide the reference, and there is a “plot_n1n100.pdf” in the results folder. This plot is not informative though, as we put only one large contig in the test dataset to reduce runtime. In the real application, with all the contigs as input, the N plot will look like what we showed in Fig S7.*

3.	The updated version is supposed to work well with Flye 2.8.3, and you said you have tested

it with a reduction on assembly errors. However in the main text still says Flye 2.8.2 (line 546) likely to avoid redoing all the analyses, that's understandable. But where are the results comparing the Flye 2.8.2 and 2.8.3 evaluation?

*Response: Thanks for pointing this out. For the error correction module of Inspector, we have updated Flye to version 2.8.3. For the assembly evaluation of HG002, we still evaluated the results from Flye 2.8.2. That’s why the Flye version stated in “whole genome de novo assembly of HG002” (line 546) is still 2.8.2. We have added the version of Flye (2.8.3) to the “Assembly error correction” part in the Methods section.*

*Regarding the results of Flye 2.8.2 and 2.8.3, we evaluated the corrected assemblies of HG002 after upgrading to Flye 2.8.3 and found an overall higher error correction rate than the previous results. As shown in Table R1, the error correction rate for structural errors was higher after upgrading to Flye 2.8.3 in 10 out of 11 assemblies. We did not include the comparison in the manuscript as we intended to emphasize the comparison between Inspector and other polishing methods, instead of the improvement of Inspector itself after upgrading packages.*
